# Advancing Tuberculosis Chemotherapy: Targeted Nanomedicines for the Mycobacterium TB Granuloma

**DOI:** 10.1002/smll.202506381

**Published:** 2025-10-30

**Authors:** Shreya Ranjith Singh, Maya Mellisa Makatini, Thashree Marimuthu, Yahya Essop Choonara

**Affiliations:** ^1^ Wits Advanced Drug Delivery Platform Research Unit Department of Pharmacy and Pharmacology School of Therapeutic Sciences Faculty of Health Sciences University of the Witwatersrand 7 York Road Parktown Johannesburg 2193 South Africa; ^2^ Wits Infectious Diseases and Oncology Research Institute Faculty of Health Sciences University of the Witwatersrand Johannesburg South Africa; ^3^ Molecular Sciences Institute School of Chemistry University of the Witwatersrand Private Bag 3 PO WITS 2050 South Africa

**Keywords:** drug delivery, granuloma, nanosystems, targeted drug delivery, tuberculosis

## Abstract

This review examines the potential of nanosystems for targeted tuberculosis (TB) therapy, focusing on biodegradable polymeric, lipid‐based, extracellular vesicles, and selected inorganic nanocarriers engineered to deliver anti‐TB drugs directly to granulomas, the hallmark of TB pathology. Both passive and active targeting strategies are discussed, emphasizing how these approaches enhance drug accumulation at infection sites to curb disease progression. Preclinical studies, including laboratory and animal models, are reviewed to assess their therapeutic impact. Although the results are promising, hurdles such as biocompatibility, regulatory constraints, and optimizing drug release still pose challenges for clinical implementation. However, rationally designed nanosystems hold significant potential for improving TB treatment outcomes. Further research is crucial for refining nanocarrier design and addressing translational hurdles. Future directions include integrating nanosystems with advanced molecular imprinting technologies (MIT) and molecularly imprinted polymer nanoparticles (MIPNPs) for enhanced granuloma targeting and controlled drug release. Additionally, immunotherapy and gene therapy offer novel adjunct strategies to boost host immunity and deliver targeted genetic interventions. These emerging approaches, combined with optimized nanosystems, have the potential to revolutionize TB management by improving drug delivery, reducing treatment duration, and enhancing therapeutic outcomes.

## Graphical abstract

This graphical abstract illustrates nanosystems used for targeted drug delivery into tuberculosis granulomas. It shows how targeting is influenced by size, pH, redox sensitivity, and surface charge. Various nanoparticles are incorporated with drugs to enhance penetration, stability, and release. This strategy minimizes side effects, improves patient compliance, and overcomes drug resistance in TB treatment. Image created in BioRender. Choonara, Y. (2025) https://BioRender.com/vdouhzs.

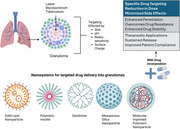

## 1 Introduction

As an infectious agent, tuberculosis (TB) continues to be the leading cause of death world‐wide, with Africa bearing a disproportionate burden accounting for over 25% of global TB‐related deaths.^[^ ^1^ ^]^ The high prevalence of latent tuberculosis infection (LTBI) in Africa significantly contributes to the continued increase in active TB cases due to the higher risk of developing the active disease. In South Africa, the situation is exacerbated by an increasing incidence of drug‐resistant TB strains, including multidrug‐resistant (MDR) and extensively drug‐resistant (XDR) TB. These resistant strains significantly complicate treatment regimens and undermine efforts to reduce TB incidence and mortality, placing a strain on public health strategies.

Granulomas are structured and organized aggregates of immune cells, primarily macrophages, which develop as a hallmark response to persistent and indigestible antigens or foreign bodies.^[^ ^2^ ^]^ These cellular assemblies encapsulate and isolate the offending stimulus, reflecting the body's endeavor to shield itself from potential harm. Although often considered a protective response, granulomas are also implicated in various diseases. However, the tightly packed structure of granulomas poses significant challenges for conventional drug delivery mechanisms. Their dense cellular configuration can act as a barrier by impeding the penetration of therapeutic agents.^[^ ^3^ ^]^ Furthermore, the internal microenvironment of granulomas, which can be characterized by varied pH levels, enzyme concentrations, and reduced blood flow, can deactivate or degrade conventional drugs before they reach their intended target. This not only diminishes the therapeutic efficacy but also necessitates higher doses with a higher chance of systemic side effects and reduced patient compliance.^[^ ^4^ ^]^ These challenges have created the need for transformative treatments through the design of innovative drug delivery systems tailored to navigate the unique structural and biochemical landscape of granulomas. Targeted drug delivery promises enhanced therapeutic outcomes by ensuring drugs are delivered directly to the granuloma to maximize efficacy and minimize collateral damage to healthy tissue.^[^ ^5^ ^]^ By leveraging advancements in nanomedicine and biotechnology, targeted systems that are activated in response to specific triggers within the granuloma can be rationally engineered. This level of precision improves treatment efficacy while enabling lower dosages, minimizing side effects, and improving patient adherence.^[^ ^6^ ^]^

Biodegradable Nanosystems offer novel ways to target specific sites, coupled with potentially reduced chances of systemic side effects. These nanosystems typically consist of lipids, polymers, or other biocompatible materials and are designed to degrade over time to lower the risk of toxicity over long‐term use and endow tailorable release mechanisms. This is especially relevant for the treatment of chronic diseases such as granulomatous conditions, including TB, where targeting systems could greatly enhance the efficiency and safety of such treatments.^[^ ^7^ ^]^

Nanosystems have been extensively explored for their potential to treat granulomas and offer distinct advantages based on their physicochemical properties. It is possible to design these nanosystems to function via passive or active targeting.^[^ ^7^ ^]^ Leveraging the Enhanced Permeability and Retention (EPR) effect, passive targeting allows for nanoparticles to preferentially accumulate in infected or inflamed tissues where the where blood vessels are more permeable. Active targeting has been primarily achieved by modifying the nanoparticle surface with ligands or antibodies that selectively bind to receptors on granuloma‐associated cells. Successful application of active targeting would result in the precise delivery of therapeutics to the site of infection or inflammation.^[^ ^8^ ^]^

In recent years, several reviews have summarized the potential of nanomaterials in TB therapy, discussing different nanosystem platforms, mechanisms, and translational challenges.^[^ ^9^ ^]^ However, these works have largely focused on general nanomedicine strategies for TB or pulmonary delivery technologies, without providing a detailed analysis of the unique pathophysiological barriers posed by granulomas and how emerging ligand‐functionalized or molecularly imprinted polymer (MIP)‐based systems can be tailored to overcome these barriers. In contrast, the present review integrates the latest understanding of TB granuloma biology with the design principles of biodegradable nanosystems, placing specific emphasis on strategies that exploit receptor‐mediated macrophage targeting, such as mannose‐functionalized and MIP‐coated nanoparticles. As such, this paper provides a comprehensive and mechanism‐driven discussion of how these nanosystems can be engineered to penetrate granulomas, remain stable within their hostile microenvironment, and release drugs in a controlled manner. Furthermore, the present review critically evaluates emerging smart‐trigger designs responsive to pH, enzymatic activity, or redox gradients and offers a forward‐looking perspective on next‐generation TB therapeutics. Owing to their small size, tunable physicochemical properties, and favorable safety profile, these biodegradable nanosystems are exceptionally well‐suited to address the complexities of granuloma treatment.^[^ ^10^ ^]^ Through an in‐depth exploration of their design, mechanisms, and applications in TB granuloma therapy, this review further highlights their diversity, advantages, and challenges, while providing insights into future directions and emerging technologies at the intersection of granuloma biology and nanotechnology.

## 2 Granuloma Formation, Pathophysiology, and Progression of *Mycobacterium tuberculosis* (*M.tb*) Infection

**Figure 1:**
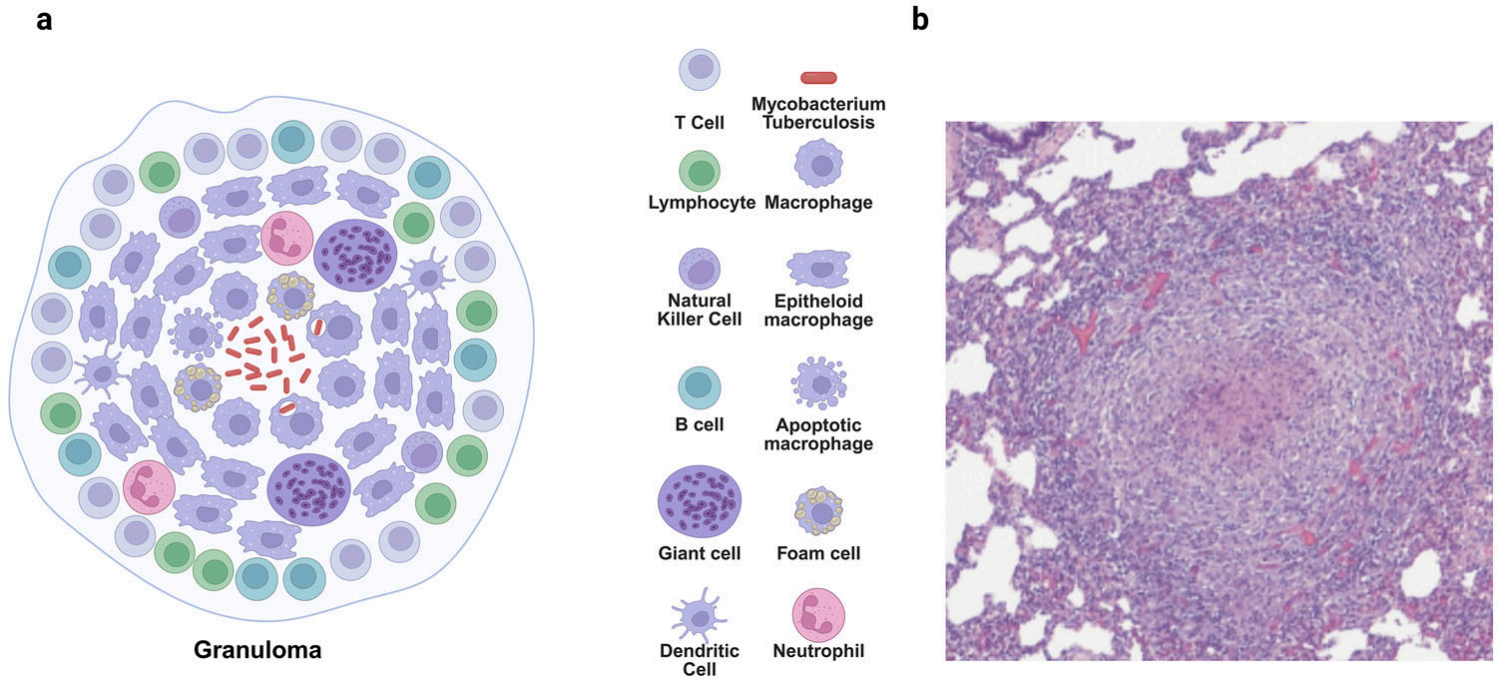
Granuloma architecture and histopathology. a) Schematic of a TB granuloma illustrating key immune cell populations and bacilli at the infection site (created in BioRender. Choonara, Y. (2025) https://BioRender.com/n55sin7). b) Representative granuloma with central necrosis from minipig lung, formalin‐fixed and stained with hematoxylin‐eosin. Reproduced under the terms of the Creative Commons Attribution (CC BY)^[^ ^12^ ^]^ license Copyright 2010, PlosOne.

A granuloma is a compact and organized immune structure composed of diverse immune cells‐ including macrophages, monocytes, dendritic cells, neutrophils, epithelioid cells, foamy macrophages, and multinucleated giant cells, and encased by a surrounding layer of lymphocytes, which imparts a solid and nodular appearance (**Figure** **1**a). The granuloma, a defining feature of tuberculosis, creates an immune microenvironment that allows for the control of the infection. Granuloma architecture varies slightly between species but generally retains defining features. In the minipig model, for instance, (Figure 1b) depicts a granuloma with a necrotic core and peripheral cellular organization, closely mirroring the histopathology of human TB. Despite this defensive role, the granuloma also offers *M.tb* a niche where it can persist, adapting the immune response to facilitate its survival without causing harm over long durations.^[^ ^11^ ^]^ Importantly, maintaining a delicate balance between pro‐inflammatory and anti‐inflammatory cytokines is essential for controlling bacterial growth during the establishment of infection.

**Figure 2:**
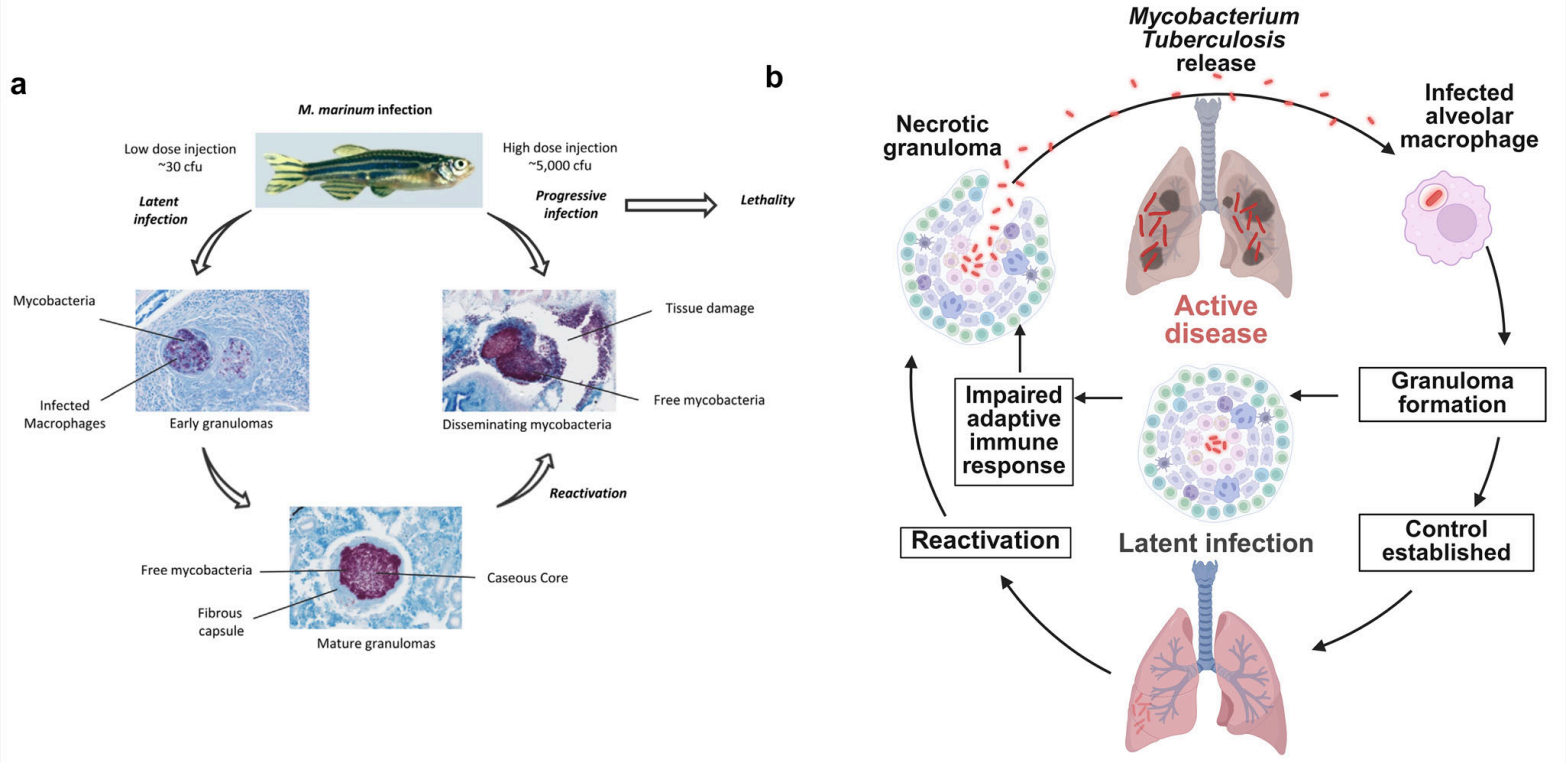
The zebrafish as a model for mycobacterial disease and TB pathogenesis. a) Adult zebrafish infected with *M. marinum* develop a latent or active progressive disease in a dose‐dependent manner: latent infection features granuloma formation across organs with maturation to caseous cores encased by a fibrous wall and an asymptomatic host, whereas reactivated or progressive infection involves granuloma disruption, rapid bacillary replication and dissemination, extensive tissue damage, and eventual mortality in most fish. Granulomas are visualized by Ziehl–Neelsen staining; mycobacteria appear as purple rods. Reproduced under the terms of the Creative Commons Attribution (CC BY 4.0)^[^ ^23^ ^]^ license Copyright 2016, Frontiers. b) Schematic of Tuberculosis pathogenesis from initial *M.tb* infection of alveolar macrophages through granuloma formation, latency, possible reactivation, and progression to active disease (created in BioRender. Choonara, Y. (2025) https://BioRender.com/xganq7a).

A study using an embryonic zebrafish model detailed the initial phases for formation of the granuloma, indicating that the bacteria replicate inside macrophages and influence the movement and recruitment of uninfected macrophages. *Mycobacterium marinum (M. marinum)*, a close relative of *M. tb* that causes cutaneous “fish‐tank granuloma” in humans, likewise replicates within macrophages and forms TB‐like lesions. With its shorter generation time and lower biosafety risk, *M. marinum* establishes the zebrafish as a practical surrogate model for TB pathogenesis (**Figure** **2**a). The presence of the bacteria also triggers epithelial cells to secrete host matrix metalloproteinase‐9 (MMP‐9), a key mediator in the pathogenesis of *M.tb*.^[^ ^13^ ^]^ Furthermore, particular bacterial elements stimulate specific immune responses, such as macrophages differentiation into foamy macrophages, associated with granuloma's necrotic regions.^[^ ^14^ ^]^

Macrophages are crucial at every stage of granulomatous inflammation, shaping the granuloma's proinflammatory milieu and driving persistent inflammation and the eventual fibrosis.^[^ ^15^ ^]^ When macrophages become infected, they trigger the release of inflammatory proteins, which attract more cells capable of phagocytosis. Even though these cells don't enhance the elimination of already engulfed mycobacteria, they contribute to the creation of granulomas.^[^ ^16^ ^]^

Dendritic cells, part of the initial responders to infection, play a significant role in immunity, engaging with bacterial components and migrating to lymph nodes to stimulate an acquired immune response.^[^ ^17^ ^]^ However, *M.tb* can interfere with this function, delaying the adaptive immune reaction necessary to halt bacterial growth. Other myeloid cell subsets also participate in granuloma formation, influencing the progression and outcomes of *M.tb*.^[^ ^18^ ^]^ Notably, the granuloma's final formation stage involves T and B cells surrounding the granuloma, solidifying its structure. The interaction of specific T cells in the lungs, producing cytokines like TNF and IFN‐γ, play a vital role in regulating bacterial growth.^[^ ^19^ ^]^ For a lasting defense against *M.tb*, a balanced immune response is vital, ensuring protection while minimizing excessive inflammation and tissue damage.

TB pathogenesis is initiated when aerosolized *M.tb* bacilli are inhaled and deposited in alveolar spaces. Resident alveolar macrophages phagocytose the bacilli, however *M.tb* employs multiple virulence strategies to inhibit phagolysosomal fusion and resist host antimicrobial mechanisms to enable survival and replication within host cells.^[^ ^20^ ^]^ This triggers a strong innate and adaptive immune response that culminates in granuloma formation. Granulomas serve a dual role: while they confine bacilli to limit spread, they also harbor persistent bacteria that may reactivate if host immunity wanes. In immunocompetent hosts, granuloma integrity is maintained, leading to LTBI, where bacilli persist without symptoms or transmissibility.^[^ ^21^ ^]^ However, in the presence of immunosuppressive conditions such as HIV co‐infection, malnutrition, or immunosenescence, granuloma structure may collapse, resulting in caseous necrosis and cavitation. This breakdown allows extracellular bacilli release into the airways that enables progression to active TB and facilitates transmission. The transition from latency to active disease is often driven by dysregulated immune signaling, where an imbalance between pro‐inflammatory and anti‐inflammatory pathways accelerates tissue damage (Figure 2b).^[^ ^22^ ^]^

## 3 Nanosystems and Targeted Drug Delivery

Targeted drug delivery to granulomas associated with *M.tb* can be effectively achieved using nanosystems, offering significant therapeutic potential. Looking though a lens of physiochemical properties of nanoparticles that includes nanoscale size, high surface area‐to‐volume ratio, and adaptable surface chemistry, researchers can design advanced drug delivery platforms specifically aimed at targeting intracellular pathogens with enhanced precision.^[^ ^24^ ^]^ These platforms demonstrate the capability to traverse granulomatous tissues and in a meticulously controlled manner deliver therapeutics. Such nanosystems hold considerable potential for augmenting the effectiveness of *M.tb* therapy by surmounting biological barriers inherent within granulomas. This facilitates the direct conveyance of anti‐TB medications to the focal point of infection, thus reducing systemic adverse effects and improving patient prognosis.^[^ ^25^ ^]^

### 3.1 Routes of Administration to Treat TB

The development of effective drug delivery strategies capable of targeting granulomas remains a critical challenge in TB therapy in light of the role of these structures in disease persistence and drug resistance. Conventional administration routes such as oral and intravenous delivery enable systemic drug distribution but often fail to achieve therapeutically relevant concentrations within granulomas. Oral administration, though convenient, is limited by degradation of drug molecules in the harsh gastrointestinal environment and variable absorption rates that leads to inconsistent and frequently suboptimal drug levels at the site of infection.^[^ ^26^ ^]^ Intravenous delivery, while bypassing first‐pass metabolism, also faces barriers to adequate penetration into lung tissue that reduces effectiveness for targeting the complex microarchitecture of granulomatous lesions.^[^ ^27^ ^]^

Pulmonary administration via inhalable formulations offers a promising alternative that provides direct access to the primary site of TB infection. Delivery systems such as dry powder inhalers and nebulizers facilitate regional targeting by depositing higher drug concentrations into granuloma‐rich lung regions.^[^ ^28^ ^]^ This organ‐level targeting not only improves local bioavailability but also reduces systemic exposure, thereby minimizing adverse effects associated with systemic therapy. Furthermore, the potential for self‐administration makes inhalable delivery particularly advantageous in low‐ and middle‐income countries where healthcare access may be limited.^[^ ^29^ ^]^

Nevertheless, regional lung targeting does not inherently guarantee specific delivery to granulomas or infected macrophages. Nanoparticles administered via the pulmonary route may distribute broadly across alveolar spaces, airway surfaces, and interstitial tissues, with only a fraction accessing the microenvironments in which *M.tb* resides.^[^ ^30^ ^]^ This limitation is particularly relevant in LTBI, where bacilli are typically sequestered deep within granulomas and are protected by dense cellular and fibrotic barriers. In such cases, pulmonary administration should be combined with rational nanosystem engineering to achieve lesion‐ or cell‐level specificity.

Several biological and physiological factors constrain the efficiency of pulmonary delivery. These include rapid mucociliary clearance and phagocytic clearance by alveolar macrophages before granuloma access, non‐uniform aerosol deposition that may limit delivery to apical lung regions that is often a predilection site for granuloma formation, and the potential for nanoparticles to remain in airway or interstitial compartments without penetrating the granuloma microenvironment.^[^ ^3^ ^]^

**Figure 3:**
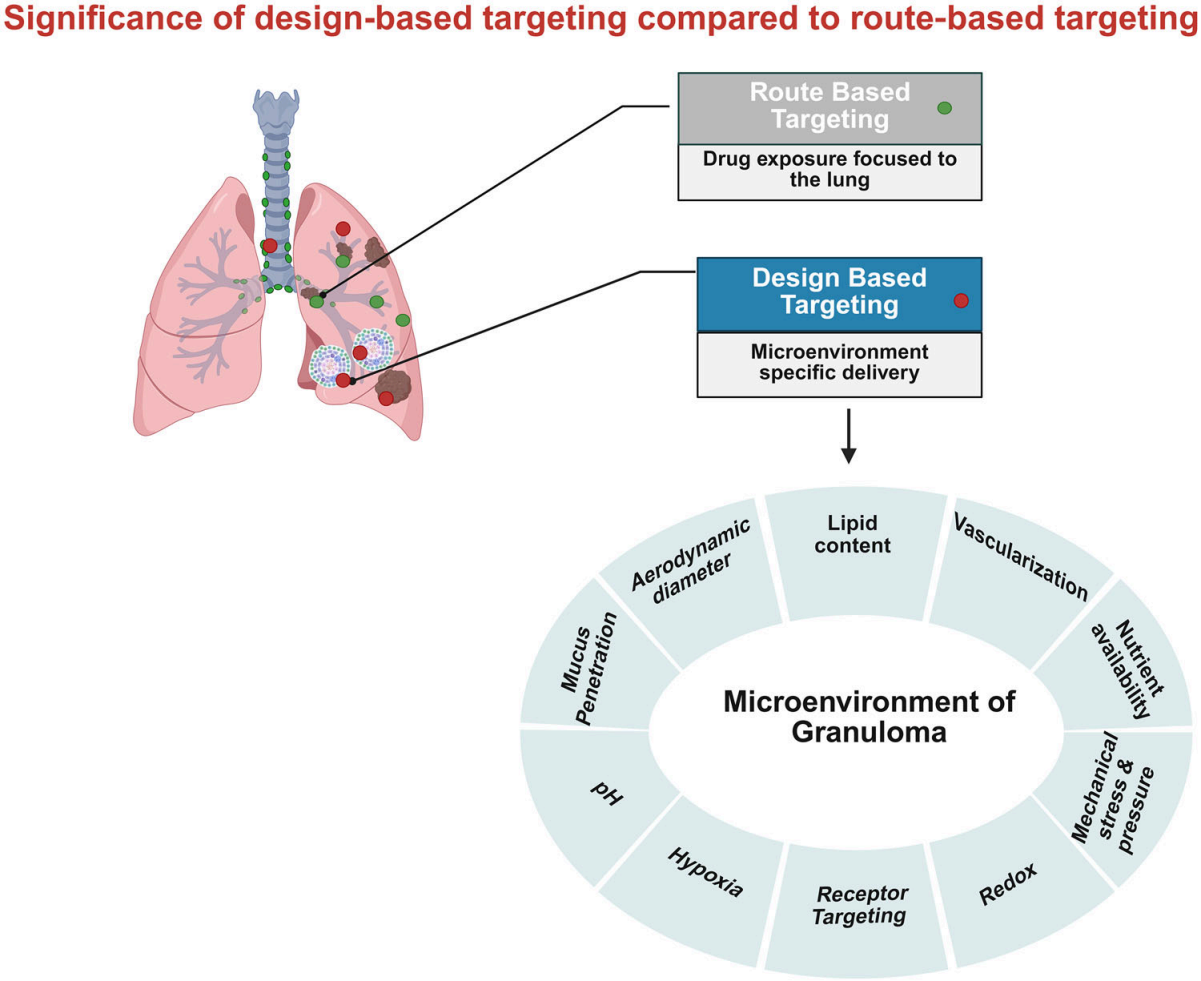
Schematic representation of pulmonary drug delivery for tuberculosis, illustrating the distinction between design‐based and route‐based targeting. Image created in Biorender. Choonara, Y. (2025) https://BioRender.com/5xhtbqy

To address these barriers, it is critical to distinguish between two complementary targeting paradigms. Route‐based targeting is achieved through pulmonary administration, which confines drug exposure predominantly to the lungs to achieve organ‐level specificity. Meanwhile, design‐based targeting is accomplished by tailoring the physicochemical attributes of nanoparticles, such as size, surface charge, and functionalization with ligands (e.g., mannose for CD206+ macrophage binding), to promote selective uptake by infected cells and enhance penetration into granulomatous lesions (**Figure** **3**).^[^ ^27^ ^]^

The integration of both approaches is recommended for optimal therapeutic efficacy in LTBI: pulmonary administration to achieve regional deposition in the lung, and design‐based nanosystem engineering to ensure microenvironment‐specific drug delivery within granulomas. This synergistic strategy holds significant promise for improving localization of therapeutics to overcome host and pathogen barriers, and for enhancing treatment outcomes in both drug‐susceptible and drug‐resistant TB.

### 3.2 Specific Granuloma Targeting Mechanisms

In targeting granulomas specifically, drug delivery strategies are designed to concentrate the active pharmaceutical ingredient (API) within the granulomatous tissue where *M.tb* tends to persist. This approach ensures an elevated API concentration at the infection site, reducing levels in other tissues and thus minimizing potential side effects.^[^ ^8^ ^]^ Granuloma‐targeted drug delivery requires a dual‐component system: one part specifically identifies and binds to cells or molecular markers unique to granulomatous tissue, while the other component initiates a therapeutic effect to either eliminate the pathogen or adjust the immune response.^[^ ^31^ ^]^ As indicated, there are two main approaches for achieving targeted delivery: passive and active targeting, each of which will be discussed in further detail.

A study by Sarathy et al. (2016) examined the challenges associated with drug delivery to *M.tb* lesions in granulomas.^[^ ^32^ ^]^ This focused on predicting drug penetration effectiveness within lesions, an essential factor for improving TB treatment outcomes. Several factors were identified as influencing drug delivery, including the physicochemical properties of the drug, granuloma architecture, and the pathophysiological conditions within the lesions, such as hypoxia. The authors recommended that a deeper understanding of drug interactions with the granuloma microenvironment could enhance optimization of therapeutic strategies, particularly for drug‐resistant TB strains. Tailored drug delivery systems could significantly improve treatment outcomes for TB by ensuring that therapeutics are delivered directly to the site of infection, which is often challenging due to the unique nature of TB lesions. These lesions, such as granulomas, create a microenvironment that limits the effective penetration of conventional drugs. By using advanced drug delivery systems such as nanoparticles or liposomes, drugs can be delivered more efficiently and in a controlled manner to these hard‐to‐reach areas. This approach aligns with the growing need for improved therapeutic methods for TB.^[^ ^32^ ^]^

### 3.3 Selection and Mechanistic Basis of Nanosystems for Tuberculosis Treatment

#### 3.3.1 Selection Criteria for Nanosystems

The rational design of nanocarriers for TB therapy necessitates optimization of interrelated physicochemical parameters that collectively determine delivery efficiency, granuloma penetration, and safety. Shape influences both pulmonary aerodynamic deposition and subsequent biological interactions. Spherical nanoparticles typically demonstrate superior macrophage internalization due to uniform surface curvature and efficient endocytosis, whereas rod‐shaped or elongated carriers may achieve deeper tissue penetration through enhanced alignment with extracellular matrix fibers.^[^ ^33^ ^]^ Surface charge is a primary determinant of electrostatic interactions with the negatively charged bacterial cell walls, granuloma extracellular matrix, and host cell membranes. Positively charged nanoparticles often show greater adhesion to granuloma surfaces and enhanced uptake by macrophages, particularly those expressing scavenger receptors, however this can also increase the likelihood of non‐specific binding to healthy tissues.^[^ ^34^ ^]^ Charge modulation strategies‐such as zwitterionic coatings or pH‐responsive cationic groups can balance targeting efficacy with off‐target risk.^[^ ^35^ ^]^ Biodegradation profile is equally critical, as it dictates clearance and long‐term safety. Polymeric nanocarriers, such as PLGA and chitosan, degrade via hydrolysis or enzymatic pathways into biocompatible monomers, while lipid‐based systems are processed through lipolytic enzymes. In contrast, inorganic platforms like mesoporous silica undergo slower hydrolytic dissolution into orthosilicic acid, necessitating careful control of particle size and surface chemistry to ensure complete elimination.^[^ ^36^ ^]^ In vivo imaging capability, achieved via incorporation of fluorescent dyes, radionuclides, or magnetic nanoparticles, enables non‐invasive biodistribution tracking and pharmacokinetic monitoring. This is particularly valuable in TB research, where quantifying granuloma penetration in preclinical models is essential for translational progression.^[^ ^37^ ^]^

#### 3.3.2 Surfactant Nanoparticle Interactions

The pulmonary environment presents unique formulation challenges due to the presence of lung surfactant that is composed primarily of dipalmitoylphosphatidylcholine (DPPC) and surfactant‐associated proteins. These components readily adsorb onto nanoparticle surfaces, altering hydrophobicity, colloidal stability, and protein corona composition. Such modifications can influence alveolar distribution, drug release kinetics, and macrophage recognition. Understanding these surfactant–nanoparticle interactions is critical for designing inhalable TB nanocarriers with preserved stability and controlled release within the alveolar space.^[^ ^38^ ^]^

#### 3.3.3 Characterization of Nanosystems for Granuloma Targeting

Effective granuloma‐targeted delivery requires comprehensive characterization at both the physicochemical and biological levels, for example:

1. Interaction with granuloma surface structures depends on electrostatic forces, hydrophobic interactions, and specific ligand–receptor recognition. Cationic nanoparticles can also exploit attraction to the anionic extracellular matrix, while hydrophobic surface domains facilitate partitioning into lipid‐rich caseous necrotic cores.^[^ ^39^ ^]^
2. Microenvironmental barriers such as hypoxia, fibrotic encapsulation, and immune cell heterogeneity can significantly hinder penetration. Particle size uniformity, ligand density, and responsiveness to granuloma‐specific stimuli (e.g., acidic pH, enzymatic activity) are therefore crucial in overcoming these barriers.^[^ ^30^ ^]^

#### 3.3.4 Mechanism of Nanosystems for M.tb Targeting

Following deposition in the lung via inhalation, nanocarriers undergo a sequential, multiscale targeting process that includes:

1. Regional targeting: Pulmonary administration delivers nanoparticles directly to the respiratory tract, increasing local drug concentration and reducing systemic exposure.^[^ ^27^ ^]^
2. Microenvironmental localization: Size and charge‐mediated tissue interactions favor accumulation in granuloma‐rich areas, aided by altered vascular permeability and extracellular matrix composition.^[^ ^40^ ^]^
3. Cellular targeting: Functionalization with ligands such as mannose enables receptor‐mediated uptake via CD206 on alveolar macrophages; alternatively, cationic surfaces facilitate charge‐driven adsorption to infected macrophages.^[^ ^41^ ^]^
4. Intracellular delivery: Endocytosed nanoparticles release their payload within phagolysosomes, directly exposing TB to high local drug concentrations.^[^ ^5^ ^]^

Each step's efficiency is dictated by nanocarrier attributes‐ surface chemistry, ligand orientation, hydrophobic/hydrophilic balance, and degradation kinetics.

## 4 Passive Targeting

This targeting method capitalizes on the inherent physio‐pathological properties of the granulomas and the drug delivery system's natural behavior. Understanding these physiopathological properties of granulomas, such as irregular blood supply, hypoxia, fibrosis, and abnormal vascular permeability that helps explain why passive targeting can be an effective strategy for delivering drugs specifically to granulomatous tissue, improving treatment outcomes for TB and other chronic infections. By understanding how these properties influence drug distribution, passive targeting can be optimized to deliver therapeutic agents directly to the site of infection, improving treatment efficacy. Studies indicate that vesicular systems like liposomes and particulate systems such as nanoparticles are designed to improve drug stability during storage and after administration, reduce unwanted side effects, and increase both drug bioavailability and its concentration at the targeted area.^[^ ^42^ ^]^

### 4.1 EPR Effect

The EPR effect, a principle of passive targeting, occurs when abnormal vasculature with enlarged inter‐endothelial gaps and impaired lymphatic drainage permits nanoparticle accumulation in diseased tissues.^[^ ^43^ ^]^ In TB granulomas, such vascular changes may enable passive drug carrier retention, potentially enhancing local concentrations and reducing systemic toxicity.^[^ ^44^ ^]^ However, the extent of this effect can vary with granuloma maturity, heterogeneity, and immune cell infiltration, and its role in TB remains less established than in cancer.

### 4.2 Particle Size

The specific size of drug delivery mechanisms such as nanoparticles plays a critical role in passive targeting. Nanoparticles can navigate through the expanded intercellular spaces in the irregular blood vessels of granulomas, enabling selective accumulation in granulomatous tissue.^[^ ^45^ ^]^ Conversely, larger particles may be restricted from entering these tissues, limiting their therapeutic efficacy. An optimal size range for nanoparticles in passive targeting is generally considered to be between 50 and 200 nm.^[^ ^46^ ^]^ These nanoparticles could penetrate the permeable blood vessels of diseased tissues while avoiding rapid renal clearance, which commonly affects particles smaller than 10 nm.^[^ ^47^ ^]^

Granulomas, like tumors, possess pathophysiological features conducive to nanoparticle accumulation, including irregular vascular architecture and impaired lymphatic drainage. These factors, combined with the optimal nanoparticle size, enhance retention within granulomas and improve local drug concentrations. Studies have shown that nanoparticles in this size range exhibit prolonged retention in granulomas, potentially improving therapeutic outcomes in TB and other granulomatous diseases by increasing drug efficacy at the site of infection while minimizing systemic toxicity.^[^ ^48^ ^]^ Furthermore, understanding the relationship between nanoparticle size and the granuloma microenvironment is crucial for optimizing drug delivery systems targeted at TB treatment.

### 4.3 Advantages and Limitations of Passive Targeting

While the EPR effect presents a promising approach for passive targeting in granulomatous diseases, several challenges remain. A key limitation is the potential for off‐target nanoparticle accumulation in tissues with similar vascular abnormalities, such as inflamed regions or tumors.^[^ ^43^ ^]^ Since these tissues also exhibit leaky vasculature, nanoparticles may inadvertently accumulate there, increasing the risk of systemic side effects and reducing treatment specificity.^[^ ^46^ ^]^ This issue highlights the need for combining passive targeting with active targeting strategies, such as ligand‐functionalized nanoparticles, to enhance site‐specific drug delivery. Passive targeting offers several advantages, including its simplicity and cost‐effectiveness. Unlike active targeting, it does not require additional modifications or ligands to guide the drug delivery system, thereby reducing formulation complexity and costs. The unique physiology of granulomas, including irregular vasculature and impaired lymphatic drainage, increases the likelihood of site specific drug accumulation, enhancing therapeutic efficacy and reducing side effects and toxicity in non‐target organs.^[^ ^49^ ^]^

Passive targeting also has notable limitations. Its efficiency can vary depending on the individual's physiological state, the nature and progression of the disease, and the heterogeneity of granulomas.^[^ ^50^ ^]^ Granulomas differ in size, vascularization, and levels of necrosis, which can influence the extent of nanoparticle penetration and retention. Furthermore, immune cells, such as macrophages and dendritic cells within granulomas, may actively clear nanoparticles through phagocytosis, thereby reducing drug availability at the site of infection.^[^ ^51^ ^]^ These inter‐patient differences complicate the predictability of nanoparticle‐based drug delivery outcomes. Off‐target accumulation remains another significant challenge, particularly in tissues with increased vascular permeability or characteristics similar to granulomas. To address these challenges, researchers are investigating advanced nanoparticle designs, including dual‐functionalized systems that combine passive EPR targeting with active targeting ligands.^[^ ^52^ ^]^ Stimuli‐responsive nanoparticles, capable of releasing their payload in response to specific environmental cues within granulomas, such as pH changes or enzymatic activity, are also being explored to enhance selective drug delivery and minimize systemic toxicity.^[^ ^53^ ^]^

### 4.4 Passive Targeted Delivery Systems: Nanosystems for In Vivo Targeting and Monitoring/Tracking of Granulomas

#### 4.4.1 Nanoparticles with Aggregation‐Induced Emission (AIE) Carrier

**Figure 4:**
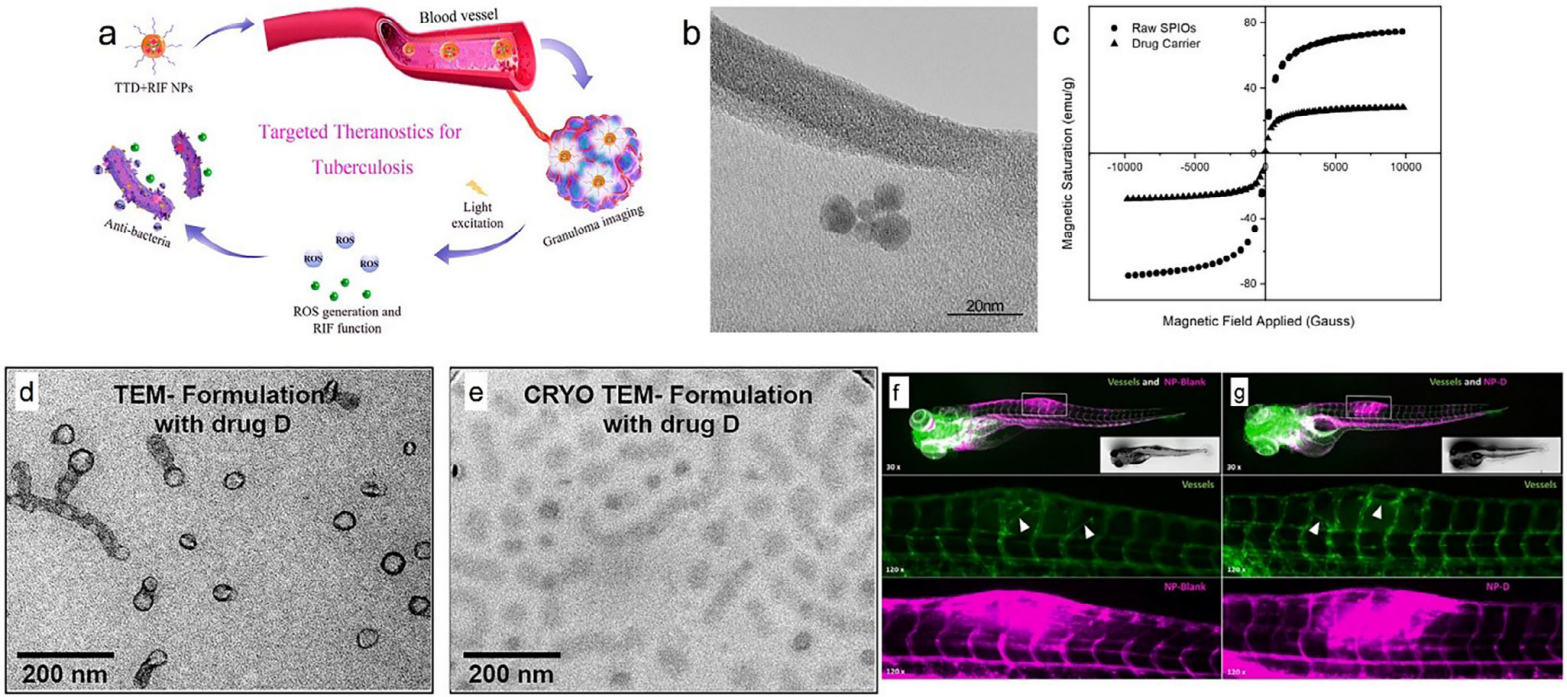
a) Nanoparticle carrier based on a amphiphilic micellar with aggregation‐induced emission (AIE) loaded with rifampicin (RIF), enabling real‐time tracking and effective drug delivery in animal reused from ref. [54] with permission Copyright 2020, American Chemical Society. Characterization of Superparamagnetic iron oxide nanoparticles. b) Transmission electron microscopy (TEM) image of spherical SPIONs, c) Hysteresis loops of the unmodified SPIONs and the drug delivery system, as measured by a Vibrating Sample Magnetometer, images b–c reused under the terms of Creative Commons CC BY 4.0 license from ref. [61] Copyright 2019, Talyor and Francis. Microscopy images of polymeric micelles, d) TEM, e) CRYO‐TEM and Representative image of a larval zebrafish 5 h post‐injection, f) with a drug‐free PeptoMicelle formulation; Top panel: 30× magnification showing fluorescent vasculature and nanoparticles (NPs); a *M. marinum* granuloma protrudes dorsally (white rectangle). Insert: corresponding bright‐field image. Middle panel: 120× magnification highlighting fluorescent vasculature (green, EGFP) in the infected neural tube region; white triangles indicate neovascularization. Bottom panel: Same field as the middle, showing fluorescent NPs (far‐red, AF647), and g) under same analysis with the drug loaded formulation. Images d–g reproduced under the terms of the Creative Commons Attribution (CC BY 4.0)^[^ ^68^ ^]^ license Copyright 2023, Elsevier.

Liao and colleagues utilized the EPR effect of nanoparticles, specifically their ability to function within the hypoxic environment of TB granulomas, to design a targeted diagnostic and therapeutic approach for TB.^[^ ^54^ ^]^ The researchers developed a carrier loaded with rifampicin (RIF) that uses aggregation‐induced emission (AIE) that was tested on laboratory animals. Post‐intravenous administration, this AIE‐based system was observed to specifically target granulomas, producing distinct fluorescent signals (**Figure** **4**a). Impressively, it could identify nascent granulomas in zebrafish embryos during the initial phases of infection and also allowed extended monitoring of the granuloma. The study demonstrated that this combined therapeutic approach had notable antibacterial results in both laboratory settings and live organisms. By leveraging light‐controlled reactive oxygen species (ROS) release and precise RIF delivery, the team successfully targeted TB.

Despite their numerous advantages, AIE nanoparticles face potential issues that may affect their performance in biomedical applications. A major concern is the stability of AIE nanoparticles over extended periods.^[^ ^55^ ^]^ Long‐term stability is essential for drug delivery systems, as nanoparticles must preserve their structural integrity and functional properties throughout storage, circulation, and within the target tissue. Instability could lead to premature aggregation or degradation of the nanoparticles, resulting in reduced therapeutic efficacy or altered optical properties.^[^ ^56^ ^]^ Environmental factors, including pH fluctuations, ionic strength, and interactions with biomolecules in vivo, can worsen this effect. To address this, surface modifications with biocompatible polymers or stabilizers are commonly employed, though these can increase formulation complexity and costs.^[^ ^57^ ^]^

Another key challenge is ensuring consistent emission in vivo across different animal models or human patients. Variability in biological environments, such as tissue composition, oxygenation levels, and immune response, can influence the photophysical properties of AIE nanoparticles. For instance, hypoxic conditions in tumors or granulomas may affect fluorescence intensity, potentially leading to inconsistent imaging results or reduced sensitivity. Moreover, differences in the biodistribution and clearance of nanoparticles across species further complicate the extrapolation of preclinical data to human applications.^[^ ^58^ ^]^ This variability underscores the need for careful in vivo characterization of AIE nanoparticles in multiple models and personalized approaches in clinical settings to ensure consistent performance.

Additionally, ensuring long‐term biocompatibility and minimizing potential toxicity remains an ongoing challenge. While AIE nanoparticles are often considered safer than traditional fluorescent dyes due to reduced aggregation‐caused quenching (ACQ) and enhanced brightness, surface coatings and degradation products may still elicit immune responses or off‐target effects in certain individuals.^[^ ^59^ ^]^ Therefore, a thorough evaluation of the pharmacokinetics, biodistribution, and immune interactions of AIE nanoparticles is critical for their successful translation from preclinical studies to clinical use.

#### 4.4.2 Amorphous Drug Nanoparticles for High‐Payload Granuloma Targeting

Nanoparticles delivered directly to the pulmonary tract can take advantage of reduced clearance rates in granulomatous tissue, as well as macrophage‐mediated uptake and trafficking, to localize near the bacilli.

A landmark example of this strategy is provided by Rudolph et al. who developed amorphous bedaquiline–pretomanid (BDQ/BTZ) nanoparticles with an unprecedented 99% drug loading ‐ the highest TB drug payload reported to date.^[^ ^30^ ^]^ The formulation process yielded stable, sub‐100 nm particles (60 ± 13 nm for BDQ; 62 ± 14 nm for BTZ) that maintained their amorphous nature, enhancing dissolution and bioavailability. Following intranasal administration to *C3HeB/FeJ* mice at doses of 6.56 mg k^−1^g BDQ and 6.72 mg k^−1^g BTZ, biodistribution studies demonstrated substantial retention within the lungs, with significantly higher drug concentrations in granuloma‐rich regions compared to the spleen and liver.

Fluorescence microscopy provided spatial evidence of nanoparticle localization, revealing deposition both in the immediate vicinity of granulomas and within infiltrating macrophage populations closely associated with *M.tb*. Although direct visualization of nanoparticles within the necrotic granuloma core was not conclusively observed, the proximity to infected macrophages strongly suggests effective engagement with intracellular bacilli in the granuloma periphery. Such passive accumulation is facilitated by the small particle size, high lipophilicity of the encapsulated drugs, and the intranasal delivery route, which maximizes deep‐lung deposition while minimizing systemic exposure.

The translational relevance of this work has been emphasized in the recent review by Carnero Canales et al., which highlights the capacity of inhalable, high‐payload nanocarriers to overcome pharmacokinetic limitations and deliver therapeutic concentrations to sites that are otherwise poorly accessible to conventional formulations.^[^ ^9^ ^]^ Collectively, these findings underscore the potential of rationally engineered, ligand‐free inhalable nanosystems to achieve targeted delivery in both active and latent TB, thereby offering a promising complement to active targeting strategies.

#### 4.4.3 Superparamagnetic Iron Oxides (SPIOs) Nanoparticles and PDLG Biocompatible Polymer

Research conducted by Arruebo et al. demonstrated that water droplets infused with superparamagnetic iron oxides (SPIOs) nanoparticles, ranging from 0.5–3.0 mm in size, can achieve nearly complete deposition in the acinar region when subjected to a magnetic field.^[^ ^60^ ^]^ Given their biocompatibility and rapid response to magnetic fields, SPIOs presented a compelling option for creating magnetically guided drug carriers.

In a subsequent study by Poh and the team, they explored the potential of SPIOs for TB treatment.^[^ ^61^ ^]^ The researchers hypothesized that encapsulating two potent TB agents, Q203 and Bedaquiline ‐ both of which inhibit bacterial oxidative phosphorylation ‐ along with a magnetic component like SPIOs into a poly(D, L‐lactide‐co‐glycolide) carrier via a single emulsion method could enhance TB treatment efficacy.^[^ ^61^ ^]^ The particles displayed a consistent size distribution ≈500 nm and possessed a magnetic saturation (28 emu g^−1^) that lay on the lower end of the typical range (Figure 4b,c).^[^ ^62^ ^]^ These particles during in‐vitro tests proved magnetically responsive, were non‐toxic to A549 epithelial cells, and effectively reduced bacterial counts of the Bacillus Calmette‐Guerin (BCG) strain. Simulations further supported the idea that using an external magnet could result in almost complete deposition in the deep lung areas.^[^ ^61^ ^]^ Hence, this approach holds promise for targeted drug delivery and can be adapted for deposition into the deep lung region of the Granuloma.

#### 4.4.4 Solid Lipid Nanoparticles Included in Microspheres

In a study, Rifabutin (RFB)‐infused solid lipid nanoparticles (SLN) were crafted to improve their delivery to the lungs. The RFB‐enriched SLN were encased in microspheres, ≈4 to 5 µm in size, using specific additives (mannitol and trehalose) via a method known as spray‐drying.^[^ ^63^ ^]^ The measured aerodynamic diameters indicated a favorable spread for targeting the alveoli. The efficacy of the dried powder in aerosol form and its in‐vitro deposition validated the microspheres' capability to penetrate the deeper regions of the lung. When these microspheres dissolve in a liquid environment, the SLNs are easily restored, preserving their original characteristics. It's anticipated that once these dry particles penetrate the deeper lung regions, the microspheres would dissolve swiftly, subsequently releasing the SLN and, consequently, the RFB. When tested in a mouse model infected with strain H37Rv, there was a notable increase in the effectiveness against *M.tb* infection compared to control groups. This suggests that encapsulating RFB in SLN offers a viable strategy for addressing TB granulomas.^[^ ^63^ ^]^

#### 4.4.5 Micelles

Rani et al. developed PEG‐PLA polymeric micelles to co‐deliver isoniazid (INH) and rifampicin (RIF), achieving a critical micelle concentration (CMC) of 8.9 ± 0.96 mg L^−1^.^[^ ^64^ ^]^ These micelles demonstrated strong anti‐*M.tb* activity and reduced haemolytic effects, indicating potential for further in vivo granuloma‐targeted TB studies.^[^ ^64^ ^]^ Biodegradable polymers like PLA and PLGA are widely used in nanoparticle systems due to their proven biocompatibility and effectiveness.^[^ ^65^ ^]^ Polymeric micelles offer several advantages, including enhanced solubility and stability. They also provide prolonged circulation time, controlled drug release, and reduced toxicity.^[^ ^66^ ^]^ Wei et al. also emphasized their safety and long in vivo circulation, supporting their use in drug delivery.^[^ ^67^ ^]^ These properties, particularly the controlled degradation of PLA blocks, make them promising for targeting TB granulomas. As shown in **Table** **1**, these nanotherapeutics enhance drug efficacy while minimizing toxicity through passive targeting approaches.

**Table 1:** Summarizing the passive delivery applications for targeted drug delivery to hinder the progression of TB.

Nanosystem	Drug	Size [nm]	Key findings	Safety	Refs.
*Polymeric Nanosystems evaluated for M*.*tb*					
TTD NPs	Rifampicin	100−120	Achieved excellent antibacterial effects for TB via light‐controlled ROS release and precise RIF delivery to granulomas.	The study lacked detailed cytotoxicity data and comprehensive safety evaluations.	[54]
Solid lipid nanoparticles (SLNs)	Rifabutin	100 and 200	SLNs in lung surfactant buffer increased lung retention following dry powder inhalation and reduced bacterial burden in the lungs, spleen, and liver of *M*.*tb* H37Rv‐infected mice.	Non‐toxic at tested concentrations, indicating a favorable safety profile for pulmonary use	[63]
PEG‐PLA polymeric micelles	Isoniazid and rifampicin	187.9 ± 2.68	PEG‐PLA micelles loaded with INH and RIF showed significantly lower MIC values for *M*. *tb* compared to the free drugs	Although detailed cytotoxicity data were lacking, PEGylated micelles generally offer reduced immunogenicity and enhanced biocompatibility	[64]
Hybrid Nanosystems evaluated against *M.tb*/acinar delivery
Poly (D, L‐lactide‐co‐glycolide) SPIONS	Q203 and Bedaquiline	500	An inhalable PDLG carrier with Q203, BDQ, and SPIONs shows strong potential for active TB treatment, ensuring high lung targeting, safe A549 cell interaction, and effective BCG‐killing	The study assessed nanoparticle cytotoxicity using A549 cells, showing cell viability and suggesting a favorable safety profile.	[61]

The zebrafish model has also been applied to assess the accumulation of micelles into granulomas. A recent study showed that polymeric micelles based on polypept(o)ides with diameters below 40 nm with varying lengths (Figure 4d,e) when injected into zebrafish larvae depicted passive accumulation in granuloma like structures (Figure 4e–g).^[^ ^68^ ^]^ The model involves injecting *M. marinum*‐into the neural tube of zebrafish larvae, effectively simulating TB granuloma like structure formation. A promising advantage is that this model can be used for pre‐screening before the use of mammalian animal models.

Nanotherapeutic strategies leverage passive targeting mechanisms to enhance drug accumulation at TB infection sites while minimizing systemic toxicity. These approaches, including magnetically guided delivery, dendritic cell‐based targeting, and controlled drug release systems, improve treatment efficacy. **Table** **2** summarizes the mechanisms, advantages, and limitations of passive targeting strategies for TB therapy.

**Table 2:** Summarizing the passive targeting strategies: Mechanisms, advantages, and disadvantages.

Nanosystem	Mechanism	Advantages	Disadvantages	Refs.
Nanoparticles with Aggregation‐Induced Emission (AIE)	AIE nanoparticles fluoresce upon aggregation, enabling drug delivery and real‐time imaging of granulomas.	Dual function: drug delivery and imaging (theranostic approach) High photostability Controlled drug release	Risk of premature aggregation. Surface modifications may induce immune responses.	[54, 55, 56]
SPIONs	Magnetically guided to granulomas, monitored via MRI.	Magnetically guided to granulomas, monitored via MRI. Magnetic targeting enhances drug accumulation MRI contrast allows non‐invasive monitoring	Stability in biological systems needs evaluation Requires external magnetic field	[60, 61]
SLNs in Microspheres	SLNs encapsulated in biodegradable microspheres for extended release.	Prolonged drug release Size dependent accumulation Stability during storage	Complexity in formulation. Stability during storage Microspheres must dissolve quickly.	[63]
Micelles	Amphiphilic molecules form carriers that encapsulate hydrophobic drugs.	Increased solubility of poorly soluble drugs Long circulation time for passive accumulation	Requires formulation optimization In vivo stability unclear. Limited clinical data on combination therapy	[64, 66, 69]

## 5 Active Targeting

Active targeting has emerged as a pivotal strategy to steer drug‐laden nanocarriers more accurately to the preferred *M.tb* locations, ensuring heightened specificity for the granuloma. This method is anchored on the engagement between specific ligands on the surface of the nanocarriers' and the target cells' receptors. Often, this tactic culminates in receptor‐mediated endocytosis, thereby amplifying the drug's concentration inside the target cells.^[^ ^70^ ^]^ Pertaining to TB, harnessing the unique receptors of the granuloma, which serve as primary host cells, can refine the precision of nanocarriers loaded with anti‐TB drugs.

### 5.1 Advantages and Disadvantages of Active Targeting in Granuloma Treatment

Utilizing active targeting in granuloma treatment ensures a more focused drug delivery. The advantages of this method include heightened treatment precision, which in turn lowers the chances of adverse effects on non‐affected sites. Moreover, it boosts the drug's impact at the actual site of the disease and might help navigate biological barriers inherent to granulomas. However, developing and producing such specialized delivery systems can be intricate and more expensive. Introducing specific ligands might also inadvertently activate the immune system. Furthermore, despite the targeting, some drug might still be taken up by non‐targeted tissues like the liver or spleen. Variations in receptor presence across patients could also impact the consistent effectiveness of this targeting strategy.

### 5.2 Ligand‐Receptor Interaction

One strategy for actively delivering drugs to granulomas involves incorporating macrophages.

Targeting specific receptors on macrophages for active delivery is theoretically feasible and enables a higher concentration of the drug to reach the granuloma, in contrast to the use of free drugs or passive targeting. The drug delivery system is modified with ligands that bind specifically to receptors on cells within the granuloma.^[^ ^52^ ^]^ Mannose and folate receptors will be discussed further.

### 5.3 Mannose Receptor

The mannose receptor (MR) is situated in alveolar macrophages and is known to interact with glycosylated lysosomal enzymes, mannose, N‐acetylglucosamine, and fucose.^[^ ^71^ ^]^ The MR receptor binds to *M.tb* due to the display of mannose and N‐acetylglucosamine on their glycoproteins.^[^ ^72^ ^]^ Generally, macrophages have mannose receptors on their surface, which can identify and attach to the mannose moiety, enhancing the cellular uptake of nanoparticles. As such, using mannose‐coated nanoformulations can be an efficient approach to actively deliver treatments to alveolar macrophages, a primary target for TB therapy.^[^ ^73^ ^]^ Enhancing drug carriers with mannose has been shown to improve their uptake by macrophages, leading to enhanced effectiveness and fewer side effects.^[^ ^74^ ^]^ This increased uptake is likely due to the specialized and selective absorption of mannose by alveolar macrophages, causing targeted accumulation of drug‐infused nanoparticles.

**Figure 5:**
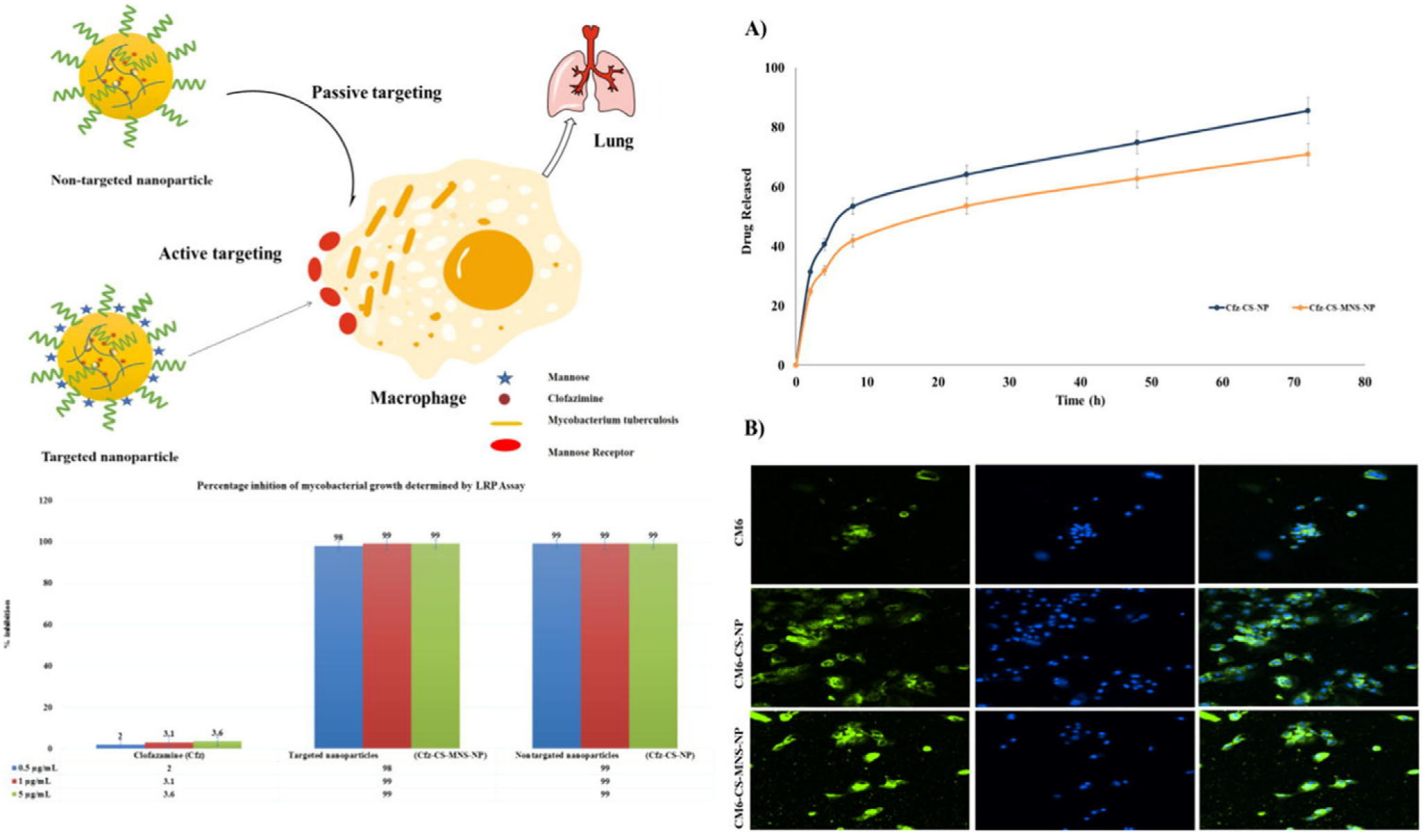
Formulation, targeting, in vitro release, uptake, and activity of clofazimine‐loaded chitosan nanoparticles A) Preparation of non‐targeted nanoparticles (Cfz‐CS‐NP), illustrated by clofazimine loading into chitosan nanoparticles (e.g., ionic gelation), targeted nanoparticles (Cfz‐CS‐MNS‐NP), showing surface mannosylation to enable receptor‐mediated targeting, proposed uptake mechanism of mannose‐receptor–targeted chitosan nanoparticles, ligand binding to CD206 on macrophages, followed by endocytosis and intracellular drug release. B) Antimycobacterial activity against *M. tuberculosis* H37Rv evaluated by the luciferase reporter phage (LRP) assay, with relative light units reflecting bacillary viability and fluorescence microscopy images of cellular uptake studies with first column FITC filter (green), followed by DAPI filter (blue) and the superimposed images thereof. Abbreviations: Cfz, clofazimine; CS, chitosan; MNS, mannosylated; NP, nanoparticle; CD206, macrophage mannose receptor; LRP, luciferase reporter phage; RLU, relative light units. Images A–B reproduced under the terms of the Creative Commons Attribution (CC BY‐NC‐ND 4.0)^[^ ^75^ ^]^ license Copyright 2020, Elsevier.

In a study conducted by Pawde et al., bioadhesive chitosan nanoparticles (NPs) with targeted receptor‐mediated properties were developed (**Figure** **5**a).^[^ ^75^ ^]^ These nanoparticles were modified with mannose on their surface to facilitate receptor‐specific drug delivery to macrophages infected with Mycobacterium (Figure 5a). Uptake studies revealed that the mannosylated nanoparticles showed significantly higher internalization efficiency than both non‐mannosylated and free mannosylated nanoparticles, thereby confirming the effectiveness of the targeted delivery method (Figure 5b).

This study shows evidence for precise targeting of the macrophage, hence leading to granuloma targeting (Figure 5b). Despite the promising potential of mannose receptor targeting, challenges exist. The expression of MR on macrophages may vary depending on the granuloma's stage and the overall immune response, which can complicate the consistency of drug delivery. Additionally, MR‐mediated uptake may be hindered by the presence of competing ligands in the granuloma, or by immune system adaptations that reduce receptor binding.^[^ ^76^ ^]^ Overcoming these challenges requires refining nanoparticle formulations to enhance specificity and reduce immune evasion.

### 5.4 Folate Receptor

Folate receptors facilitate the internalization of folic acid derivatives into cells through endocytosis. Among the different folate receptor isoforms, folate receptor β is mainly present on the surface of activated macrophages involved in inflammation and autoimmune disorders, as well as on tumor‐associated macrophages (TAMs).^[^ ^63^ ^]^ As a result, both folic acid and its derivatives have become sought‐after ligands for the targeted delivery of diagnostic and therapeutic agents to cancer cells, TAMs, and inflammatory disease‐associated macrophages.^[^ ^77^ ^]^

In the fight against TB, leveraging the folate receptor as a target has been largely overlooked. Shah and his team recently made strides in this area, crafting a nanoemulsion loaded with the anti‐TB drug RIF, designed for deep lung delivery. This formulation, coated with chitosan‐folate, showed enhanced cellular uptake, especially when compared to versions without folate.^[^ ^78^ ^]^ In addition, it demonstrated better drug retention in the lungs and reduced levels in the bloodstream. A comparable method was effectively employed, where SLNs were coated with folate‐attached chitosan to target lung tissues through inhalation. However, the primary aim of this research centered on treating lung cancer rather than TB.^[^ ^79^ ^]^ Another research approach used folate in dual capacities: as a targeting mechanism and as a component of a liquid‐crystalline folate nanoparticle structure for RIF delivery. RIF intercalated within the ordered folate stacks of the NPs and an efficient RIF loading was achieved with sustained release.^[^ ^80^ ^]^ While folate receptor targeting holds promise, there are several challenges in the context of TB treatment. Folate receptor β is often overexpressed in inflammatory conditions, but its expression can be inconsistent depending on the immune environment within granulomas. This variability may limit the effectiveness of folate‐based drug delivery systems. Furthermore, folate receptors are not exclusive to TB‐infected macrophages and can also be found on other cells, such as those in tumor tissues, which could lead to off‐target effects and unintended drug accumulation.^[^ ^5^ ^]^ To improve specificity, further research is needed to optimize the ligand‐receptor binding process and minimize potential side effects.

### 5.5 Utilizing Dendritic Cells

Dendritic cells (DCs) play a pivotal role in initiating CD41 and CD81 T‐cell reactions specific to Mycobacterium avium.^[^ ^81^ ^]^ Renowned as highly effective antigen‐presenting cells, they surpass other cell types in triggering primary immune responses. Often, they are among the initial cells to detect a pathogen and can assimilate and process the pathogen's antigens before moving to granulomas. Mycobacterium avium complex (MAC) is acknowledged for inducing lung diseases in humans. Its engagement with airway lining cells and alveolar macrophages probably marks crucial stages in MAC's disease mechanism, resulting in persistent airway infections, granulomatous inflammation, and eventually bronchiectasis.^[^ ^82^ ^]^ Research by Montes‐Worboys et al. explored the potential use of DCs loaded with a luminescent dye‐labeled antibiotic for treating MAC. By leveraging the capability of DCs to home in on granulomas, penetrate them, and target active M. avium populations while transporting concentrated intracellular drug loads, this approach could enable localized treatment while minimizing systemic toxicity.^[^ ^83^ ^]^

Using DCs to deliver amikacin in vivo capitalizes on their “memory” properties, allowing targeted drug delivery to specific locations. This strategy is novel as it leverages the capacity of dendritic cells (DCs) to recognize pathogens and facilitate antigen presentation to T cells. DCs have been extensively utilized in cancer immunotherapy and vaccine development, where activated DCs have been shown to elicit both immune responses and clinical benefits in select patients with advanced malignancies. A similar principle can be adapted for the treatment of infectious diseases, harnessing the unique properties of DCs to enable precise and targeted therapeutic interventions.

### 5.6 Stimuli‐Responsive Systems

Within the granuloma, the microenvironment can differ from the surrounding tissues in terms of pH, enzymatic activity, and other factors. By harnessing these unique characteristics, responsive drug delivery systems can be developed to specifically target granulomas.^[^ ^25^ ^]^

### 5.7 pH‐Sensitive Systems

Owing to the interactions of immune cells and infectious agents, the pH level within granulomas can vary from adjacent tissues. Some drug delivery techniques take advantage of this by employing materials that react to pH fluctuations. Zhang and colleagues developed a pH‐responsive drug delivery system made of polymeric micelles loaded with an anti‐inflammation agent and coated with lung targeted ligands to target inflammatory lungs for Acute Lung Inflammation (ALI) therapies.^[^ ^84^ ^]^

Zhang and colleagues developed pH‐responsive polymeric micelles loaded with an anti‐inflammatory agent and coated with lung‐targeting ligands.^[^ ^84^ ^]^ The NP has a core made of an acid‐sensitive material called poly(β‐amino esters) (PAE), which enabled drug loading and controlled release.^[^ ^84^ ^]^ The surface of the particle features a combination of PEG and biotin, which helps in targeting the lungs and ensures the nanoparticle remains in the bloodstream for longer. Research simulations indicate that these particles can be coated with anti‐ICAM‐1 antibodies to improve lung targeting. While this approach effectively targets inflamed lungs for Acute Lung Inflammation (ALI) therapy in mice, its overall efficacy may depend on the precise tuning of pH responsiveness and the targeting specificity of the ligands, which should be critically evaluated in further studies. Their studies reveal the rational design of nanotherapeutics for improved therapy of ALI and hence the principles could be used for targeting granuloma inflammation.

Recent advancements in drug delivery systems, such as liposome‐in‐hydrogel techniques, show potential for local bone TB treatment, using liposomes with entrapped isoniazid (INH) for targeted delivery and alveolar stabilization. Studies on dry powder inhalers (DPIs) with ligand‐anchored pH‐sensitive liposomes for INH and ciprofloxacin demonstrate effective lung accumulation, offering a promising approach for treating pulmonary TB. Such liposomal carriers improve anti‐tuberculosis drug (ATD) targeting in the lungs, enhancing therapeutic efficacy and offering an alternative to traditional TB treatment.^[^ ^85^ ^]^ Despite their potential to target the acidic environments of TB‐infected macrophages, pH‐sensitive drug delivery systems face notable challenges. Premature drug release in systemic circulation, caused by insufficient stability, can diminish therapeutic outcomes. Patient variability, such as altered physiological pH due to comorbidities, further complicates controlled drug delivery. Moreover, high production costs and the technological complexity required for scalable manufacturing limit accessibility, especially in low‐resource settings. Overcoming these limitations is crucial for making pH‐sensitive systems viable in practical TB therapy applications.^[^ ^25^ ^]^

### 5.8 Enzyme‐Sensitive Systems

Granulomas contain elevated levels of specific enzymes.^[^ ^86^ ^]^ This characteristic can be exploited to achieve targeted drug release. The role of Matrix Metalloproteinases (MMPs) is critical in lung function and disease, often contributing to tissue damage and inflammation‐associated lung conditions. They are especially active in degrading the extracellular matrix and be notably present during granuloma formation.^[^ ^87^ ^]^ Some studies advocate a novel approach to TB treatment, suggesting that inhibiting MMP activity (using agents like Doxycycline or Marimastat) can hinder matrix destruction, thereby reducing granuloma development and bacterial presence. This could subsequently decrease TB‐related complications and death rates.^[^ ^88^ ^]^ In line with this, experiments with mice deficient in MMP‐9 have indicated a reduction in macrophage attraction to the lungs and smaller granuloma formations.^[^ ^89^ ^]^ Moreover, research by Sabir's team indicated that using Marimastat to inhibit MMPs led to a decline in both granuloma formation and bacterial presence during *M.tb* infection, reinforcing the potential of MMP‐focused strategies as supplementary treatments for TB.^[^ ^90^ ^]^

### 5.9 Redox‐Responsive Systems

In the realm of drug delivery, redox‐responsive mechanisms capitalize on the imbalance of redox homeostasis in disease states. Granulomas typically display an intensified oxidative stress, resulting from the persistent efforts of immune cells to combat the underlying pathogens.^[^ ^91^ ^]^ These systems have linkages or structures that react to redox conditions. In typical situations, these linkages ensure the drug remains confined inside the delivery vehicle. However, in areas with increased redox activity, such as granulomas, the linkages break, leading to the drug's release.^[^ ^92^ ^]^ Polymers like disulfide‐containing poly(ethylene glycol) (PEG) derivatives can be sensitive to the redox environment. The disulfide bonds in these polymers are stable under normal conditions but get cleaved in the presence of reducing agents like glutathione, which is found in elevated concentrations in diseased cells.^[^ ^4^ ^]^ Researchers have developed a target‐specific drug delivery system by utilizing the elevated levels of glutathione (GSH) commonly overexpressed in the cytoplasm of cancer cells. This strategy offers precise drug delivery and robust therapeutic effects, providing redox‐sensitive drug delivery systems with several key advantages. In the absence of additional endogenous or exogenous stimuli, this approach can achieve optimal outcomes, including minimal drug leakage in the bloodstream, targeted delivery to tumors, and rapid drug release.^[^ ^93^ ^]^

**Figure 6:**
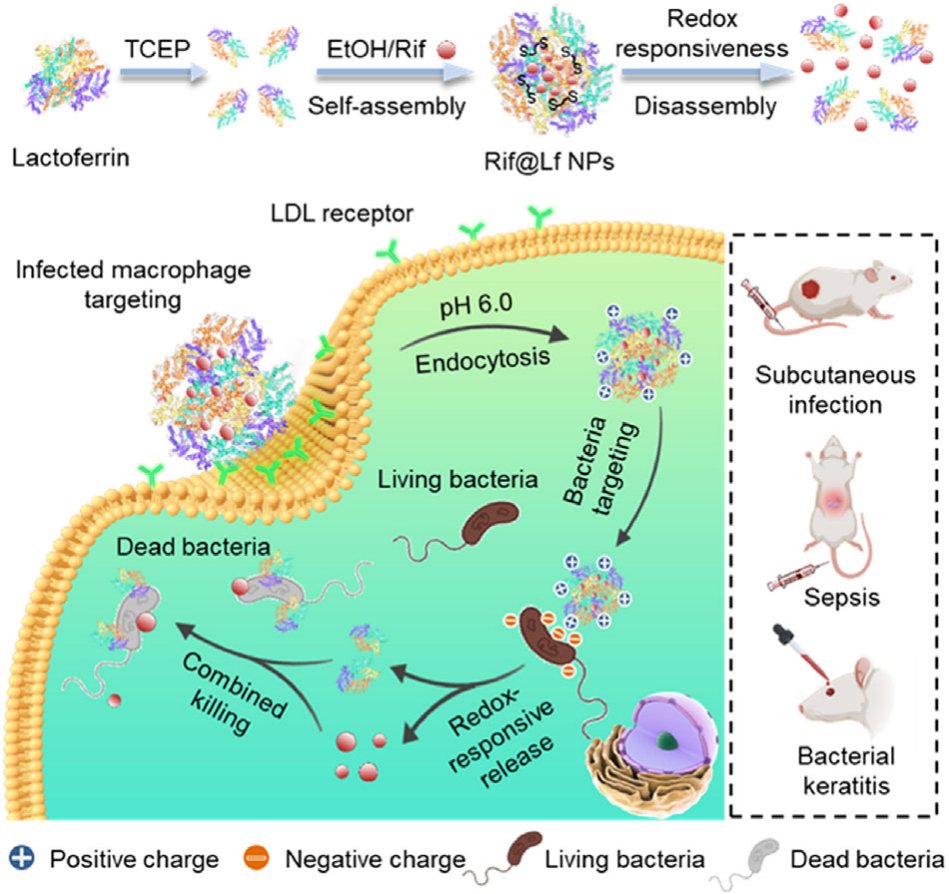
Rifampicin‐loaded lactoferrin nanoparticles (Rif@Lf NPs) for targeted intracellular antimycobacterial therapy: formation and redox‐responsive disassembly of Rif@Lf NPs, lactoferrin is reduced with TCEP to generate free thiols, co‐assembled with rifampicin in ethanol to form Rif@Lf NPs, and subsequently disassembles in an intracellular reducing milieu (e.g., glutathione) to release rifampicin and schematic of Rif@Lf NPs as a platform enabling targeted elimination of intracellular bacteria. Abbreviations: Rif, rifampicin; Lf, lactoferrin; NPs, nanoparticles; TCEP, tris(2‐carboxyethyl)phosphine; EtOH, ethanol; GSH, glutathione. Image reproduced under the terms of the Creative Commons Attribution (CC BY‐NC‐ND 4.0)^[^ ^94^ ^]^ license Copyright 2024, Elsevier.

Recent advances have extended this approach beyond oncology into infectious disease applications. For example, Wang et al. (2024) developed lactoferrin nanoparticle assemblies loaded with rifampicin (Rif‐Lf NPs) that combine macrophage‐targeted uptake with redox‐triggered drug release in infected macrophages (**Figure** **6**). This dual‐functional system enhances antibiotic accumulation within intracellular infection sites such as TB granulomas, while simultaneously benefiting from lactoferrin's inherent antimicrobial and immunomodulatory properties (Figure 6). The result is a synergistic enhancement of antibacterial efficacy in relevant infection models (Figure 6) highlighting the potential for redox‐responsive designs in TB therapy.^[^ ^94^ ^]^

Beyond disulfide linkages, other chemistries such as diselenide and tetrasulfide bonds, redox‐labile prodrug linkers, and thioketal cross‐linkers have been successfully integrated into micelles, nanogels, and polymer–drug conjugates. These systems provide rapid cleavage and controlled release under reducing conditions, offering promising adaptability for granuloma‐targeted TB treatment.^[^ ^95^ ^]^ By tailoring the carrier's sensitivity to the unique redox gradients within TB lesions, such systems can achieve precise spatiotemporal control of drug delivery, improving therapeutic outcomes while reducing systemic exposure.

### 5.10 Utilizing Pathogen‐Specific Antigens

Utilizing pathogen‐specific antigens for targeted drug delivery capitalizes on the immune response's specificity against infectious agents.^[^ ^96^ ^]^ In infectious granulomas, *M.tb* expresses specific antigens that the host immune system recognizes. By linking these antigens or their mimics to drug delivery systems, it becomes possible to direct therapeutic agents specifically to the sites of infection.^[^ ^97^ ^]^ Targeting drugs using antigens specific to pathogens capitalizes on the immune system's ability to precisely respond to infectious agents.^[^ ^98^ ^]^ *M.tb* granulomas present distinct antigens that are identified by the host's immune defenses. By attaching these antigens or their analogs to drug carriers, one can accurately steer therapeutic agents directly to infection sites.

In a study conducted by Xu et al., 2017, they investigated the generation of monoclonal antibodies (mAbs) against Ag85A Antigen of *M.tb*.^[^ ^99^ ^]^ The *M.tb* Ag85 complex has been shown to induce a robust humoral and cell‐mediated immune response.^[^ ^100^ ^]^ Furthermore, the elevated levels of serum antibodies against the Ag85 complex in patients with active tuberculosis provide additional evidence suggesting that the Ag85 complex may serve as a potential diagnostic marker.^[^ ^101^ ^]^ In this study, mAbs were generated against the recombinant Ag85A protein, demonstrating strong reactivity with both recombinant and endogenous Ag85A. These antibodies show significant potential as valuable tools for advancing diagnostic techniques and drug development for *M.tb*.^[^ ^99^ ^]^

This concept can be used for future works of targeting the granuloma by utilizing antibodies that target specific antigens of the granuloma. These antibodies can be conjugated with drugs or therapeutic agents. Once the antibody binds to the target antigen, the drug can be released into the immediate environment, acting against the pathogen or modulating the host response. Thus, resulting in selective targeted delivery. Despite these advancements, further studies are necessary to validate the efficacy of antigen‐based targeting systems in vivo. For instance, preclinical studies in animal models could provide insights into biodistribution, targeting efficiency, and therapeutic outcomes. Future work should also explore the therapeutic potential of other antigens, such as ESAT‐6, CFP‐10, or MPT64, which have demonstrated immunogenicity and diagnostic relevance in TB.^[^ ^98^ ^]^

Additionally, antigen‐based therapies could be integrated with existing TB regimens. By conjugating antibodies targeting granuloma‐specific antigens with conventional TB drugs like rifampicin or pyrazinamide, these approaches could enhance drug accumulation at the infection site while minimizing systemic side effects.^[^ ^102^ ^]^ This integration offers the potential to improve treatment efficacy, shorten treatment duration, and address challenges such as drug‐resistant TB and latent infections. The application of antigen‐specific targeting systems, particularly those utilizing monoclonal antibodies, represents a promising avenue for TB management. When combined with current therapeutic frameworks, these innovations could significantly advance the precision and effectiveness of TB treatment

## 6 Therapeutic Modalities Enabled by Nanocarriers

### 6.1 Oxygen‐Targeted Sonodynamic Therapy for Tuberculous Granulomas via Catalase‐Loaded Nanoplatform

One of the main challenges in effectively treating TB lies in the heterogeneous microenvironment of TB granulomas, which are structured, immune‐formed clusters of immune cells, bacteria, and necrotic tissue.^[^ ^103^ ^]^ These granulomas vary significantly in oxygenation, cellular composition, and pH, creating a diverse set of conditions that hinders drug penetration and efficacy. The central regions of granulomas are often hypoxic, acidic, and nutrient‐poor, providing a unique environment where *M.tb* persist in a dormant, drug‐tolerant state, effectively evading immune detection and resisting antibiotics.^[^ ^99^ ^]^ This hypoxic and acidic core is surrounded by a more oxygenated outer layer with active immune cells, which can create barriers to drug diffusion, especially for drugs that rely on oxygen for effective bactericidal action. Additionally, the presence of necrotic tissue in granulomas impedes immune cells from reaching and eliminating *M.tb* effectively.^[^ ^2^ ^]^ This complex structure and variable environment within granulomas allow *M.tb* to survive for extended periods, often resulting in latent infections and complicating treatment, especially in cases of drug‐resistant TB. Targeted therapies that can address these diverse microenvironments, such as oxygen‐enhancing strategies and sonodynamic therapies, are being investigated to potentially overcome these barriers and improve treatment efficacy in TB granulomas.^[^ ^104^ ^]^

**Figure 7:**
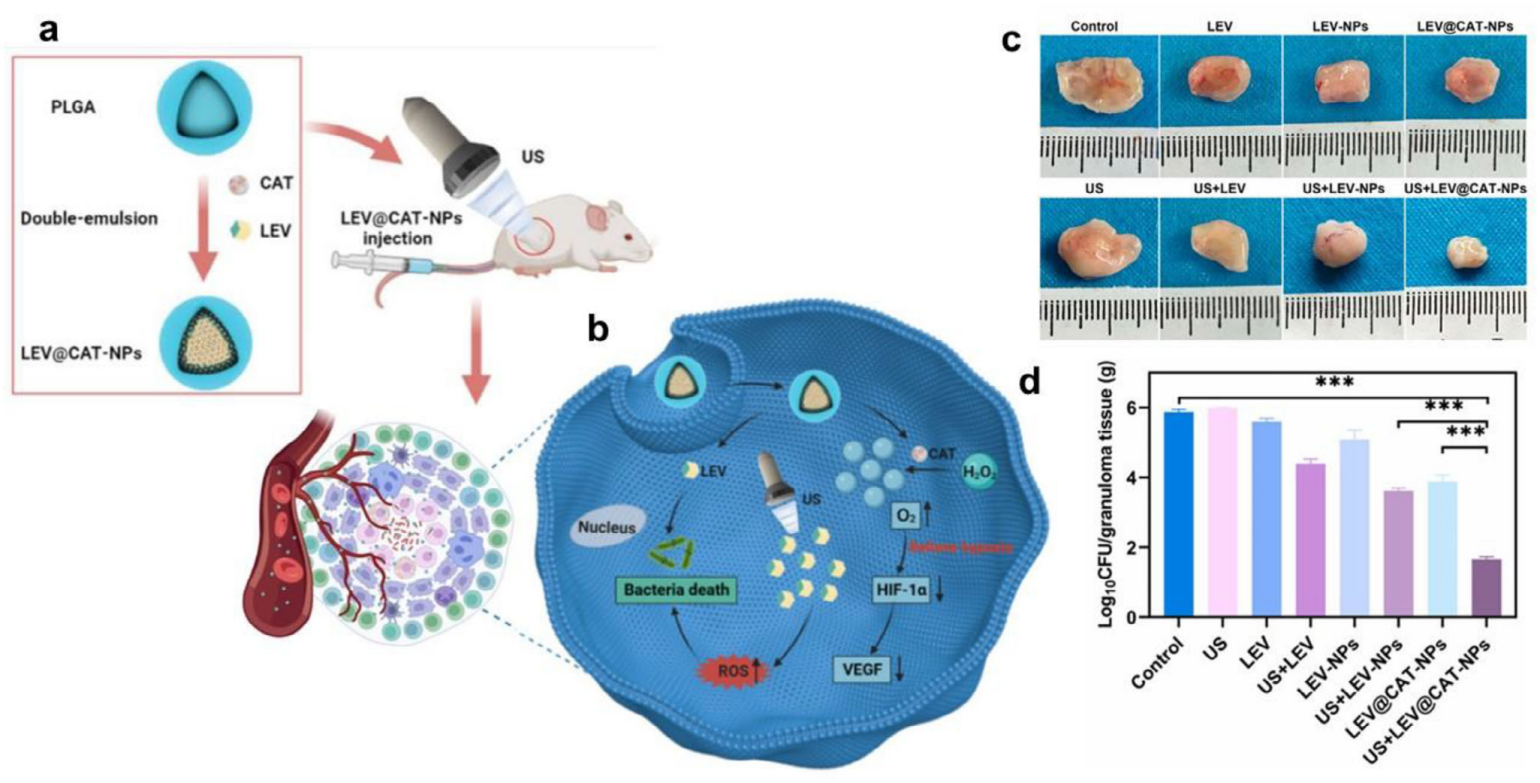
Ultrasound‐mediated LEV@CAT‐NPs for granuloma‐targeted therapy. a) Nanomaterial schematic of LEV@CAT‐NPs showing levofloxacin‐loaded, catalase‐integrated nanoparticles. b) Targeting mechanism: proposed ultrasound‐activated pathway enhancing lesion accumulation, tissue penetration, and intracellular delivery within BCG‐infected granulomas. c) Representative photographs of isolated granulomatous tissues 14 days after treatment with the indicated modalities. d) Colony‐forming unit (CFU) analysis of homogenized granulomatous tissues collected 14 days post‐treatment, comparing bacterial burden across modalities. Abbreviations: LEV, levofloxacin; CAT, catalase; NPs, nanoparticles; BCG, Bacille Calmette–Guérin; US, ultrasound. Images a–d reproduced under the terms of the Creative Commons Attribution (CC BY‐NC 3.0)^[^ ^104^ ^]^ license Copyright 2023, Dovepress Taylor and Francis Group.

Hu and colleagues explored a strategy targeting hypoxia to enhance TB treatment using ultrasound‐driven PLGA nanoparticles loaded with levofloxacin (LEV) and catalase (CAT).^[^ ^104^ ^]^ These nanoparticles, created through double emulsification, demonstrated oxygen production, biocompatibility, and safety in a mouse model of subcutaneous TB granulomas induced by Bacille Calmette‐Guérin (BCG) (**Figure** **7**a,b). Ultrasound‐enhanced nanoparticles significantly reduced bacterial presence, improved oxygen levels, reduced inflammation, and promoted tissue repair. Additionally, they lowered inflammatory cytokines and suppressed the expression of hypoxia‐inducible factor 1α (HIF‐1α) and vascular endothelial growth factor (VEGF), suggesting their potential in targeting bacterial infections and improving granuloma outcomes. These effects were evident in representative granulomatous tissues isolated 14 days after treatment (Figure 7c) and further confirmed by CFU analysis of homogenized tissues, which showed a marked reduction in bacterial burden across modalities (Figure 7d).

### 6.2 Host‐Directed Therapy: Granuloma‐Targeted Approaches to Modulating Angiogenesis

Host‐directed therapy (HDT) focuses on altering the host's immune system or physiological mechanisms to enhance infection treatment, including *M.tb*. In granulomas, which are formed as a result of TB infection, the regulation of angiogenesis‐new blood vessel growth‐can significantly impact the disease's progression and treatment outcomes.^[^ ^69^ ^]^ Granulomas often exhibit varying levels of vascularization, with some areas experiencing insufficient blood flow and hypoxia, while others may develop excessive new vessels. By either promoting or inhibiting angiogenesis, granuloma‐targeted therapies can help balance the blood supply, thereby improving immune cell infiltration and drug delivery in under‐oxygenated regions or reducing inflammation in over‐vascularized areas.^[^ ^105^ ^]^ This approach aims to optimize the granuloma microenvironment to improve treatment efficacy for TB by enhancing immune responses and promoting tissue repair.

### 6.3 Uptake of Biomimetic Nanovesicles by Granuloma for Photodynamic Therapy of Tuberculosis

**Figure 8:**
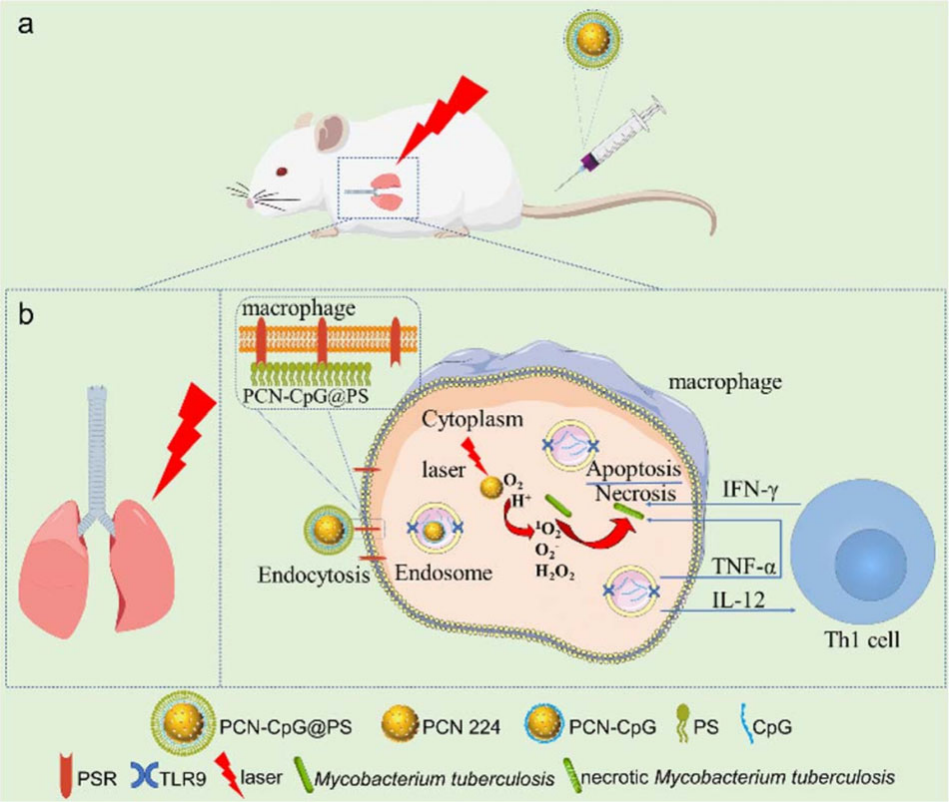
a) Schematic of in vivo administration of macrophage‐targeted nanoparticles for combined photodynamic immunotherapy, b) proposed specific mechanism by which the nanoparticles induce photodynamic and immunotherapeutic effects in macrophages. Reproduced under terms (CC BY‐NC 3.0)^[^ ^106^ ^]^ Copyright 2023, Royal Society Chemistry.

Photodynamic therapy (PDT) is a promising method for targeting *M.tb* using light‐activated photosensitizers that generate ROS. However, delivering these photosensitizers effectively to TB granulomas is challenging due to their dense structure and hypoxic conditions. Recent studies show that biomimetic nanovesicles can enhance drug delivery by up to 60%, improving the precision and efficiency of treatment. These nanovesicles, designed to mimic natural cell membranes, help overcome barriers in granulomas, allowing for better drug penetration and controlled release. A study presents a dual‐functional nanoplatform for TB therapy, utilizing phosphatidylserine‐coated nanoparticles to achieve targeted delivery to macrophages within granulomatous lesions.^[^ ^106^ ^]^ The nanoparticles, designed to mimic apoptotic bodies, enhance cellular uptake by infected macrophages (**Figure** **8**a). Upon light irradiation, the photosensitizer generates reactive oxygen species, facilitating intracellular bacterial clearance through photodynamic effects (Figure 8b). Concurrently, the CpG oligodeoxynucleotides serve as immunostimulatory agents, activating innate immune pathways. The combination of targeted photodynamic therapy and immune activation provides a synergistic strategy for effectively treating persistent and drug‐resistant *M.tb* infections.

**Figure 9:**
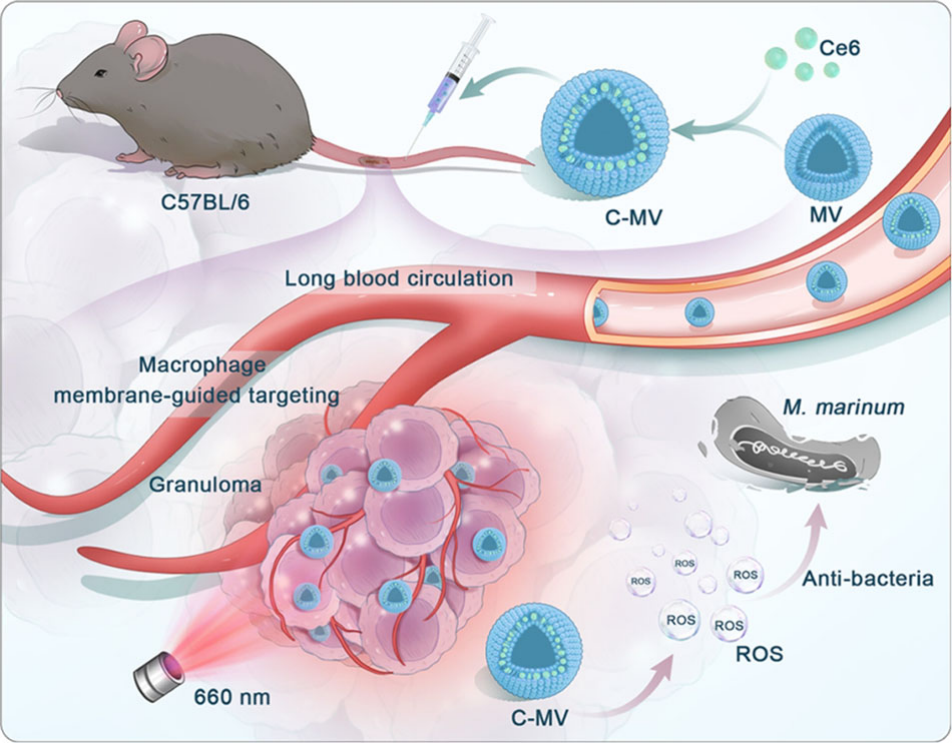
Macrophage‐guided nanoparticles for granuloma‐targeted photodynamic therapy in Tuberculosis. Reproduced under creative commons (CC BY‐NC‐ND 4.0)^[^ ^107^ ^]^ Copyright 2025, American Chemical Society.

In a study by Wang et al., a biomimetic nanovesicle, was developed to improve PDT for TB. Chlorin e6 (Ce6), a photosensitizer, was incorporated into the nanovesicles, facilitating both photodynamic bacterial killing and fluorescence imaging of granulomas.^[^ ^107^ ^]^ When injected intravenously, the nanovesicle exhibited prolonged circulation times due to the protective macrophage membrane, which shields them from rapid clearance (**Figure** **9**). These nanoparticles were successfully directed to granulomas, where they delivered targeted therapy. Photosensitizer generates ROS upon light activation, leading to M. marinum death. The nanovesicle demonstrated excellent biocompatibility, long circulation time, and significant inhibition of bacterial proliferation, offering a novel approach to targeted TB therapy, particularly in cases of drug resistance.

Recent advances have further refined this strategy by incorporating functional modifications to enhance lesion specificity. Li et al. (2024) reported activated macrophage–membrane‐coated nanoparticles, in which activation upregulated chemokine receptors and adhesion molecules, resulting in superior homing to granulomatous tissue and enabling photothermal ablation of infected lesions.^[^ ^108^ ^]^ This represents a clear shift from passive immune‐camouflage to active, receptor‐mediated targeting.

Macrophage‐derived biomimetic nanoparticles, such as those developed by Wang et al. (2024), leverage the natural targeting capabilities of macrophage membranes to enhance delivery to pathogen‐rich lesions. In their study, macrophage membrane‐cloaked aggregation‐induced emission nanoparticles (PN‐AIE MØs) retained key membrane proteins from RAW 264.7 cells, enabling them to effectively adsorb vaccinia virus particles and target pustular lesions in an Mpox model through membrane‐mediated homotypic recognition and improved retention in target tissues.^[^ ^109^ ^]^ Analogously, in the context of TB, granulomas are rich in macrophages and inflammatory markers. Biomimetic vesicles or nanovesicles derived from macrophage membranes can harness these same targeting mechanisms‐facilitated by integrins, selectins, and adhesion molecules such as LFA‐1 or PSGL‐1‐for enhanced accumulation in inflammatory and granulomatous tissues. Indeed, macrophage membrane‐coated nanoparticles have shown promising targeting and biocompatibility profiles in models of inflammation and atherosclerosis.^[^ ^110^ ^]^ Thus, incorporating macrophage membrane‐derived vesicles into TB‐targeted nanotherapies may greatly enhance selective delivery to granuloma‐associated macrophages and mycobacteria, improving both therapeutic index and lesion‐specific uptake through a biomimetic “homing” strategy.

In parallel, macrophage‐derived extracellular vesicles (EVs) are emerging as intrinsic nanocarriers with natural tropism toward TB lesions. Sun et al. (2024) comprehensively reviewed their potential for drug and diagnostic payload delivery, highlighting the influence of surface protein composition on tissue specificity and immune modulation.^[^ ^111^ ^]^ Furthermore, EVs from helminth‐antigen‐exposed macrophages have been shown to suppress *M.tb* growth in vitro by modulating pro‐inflammatory cytokine pathways^[^ ^112^ ^]^ underscoring their therapeutic potential in TB.

**Figure 10:**
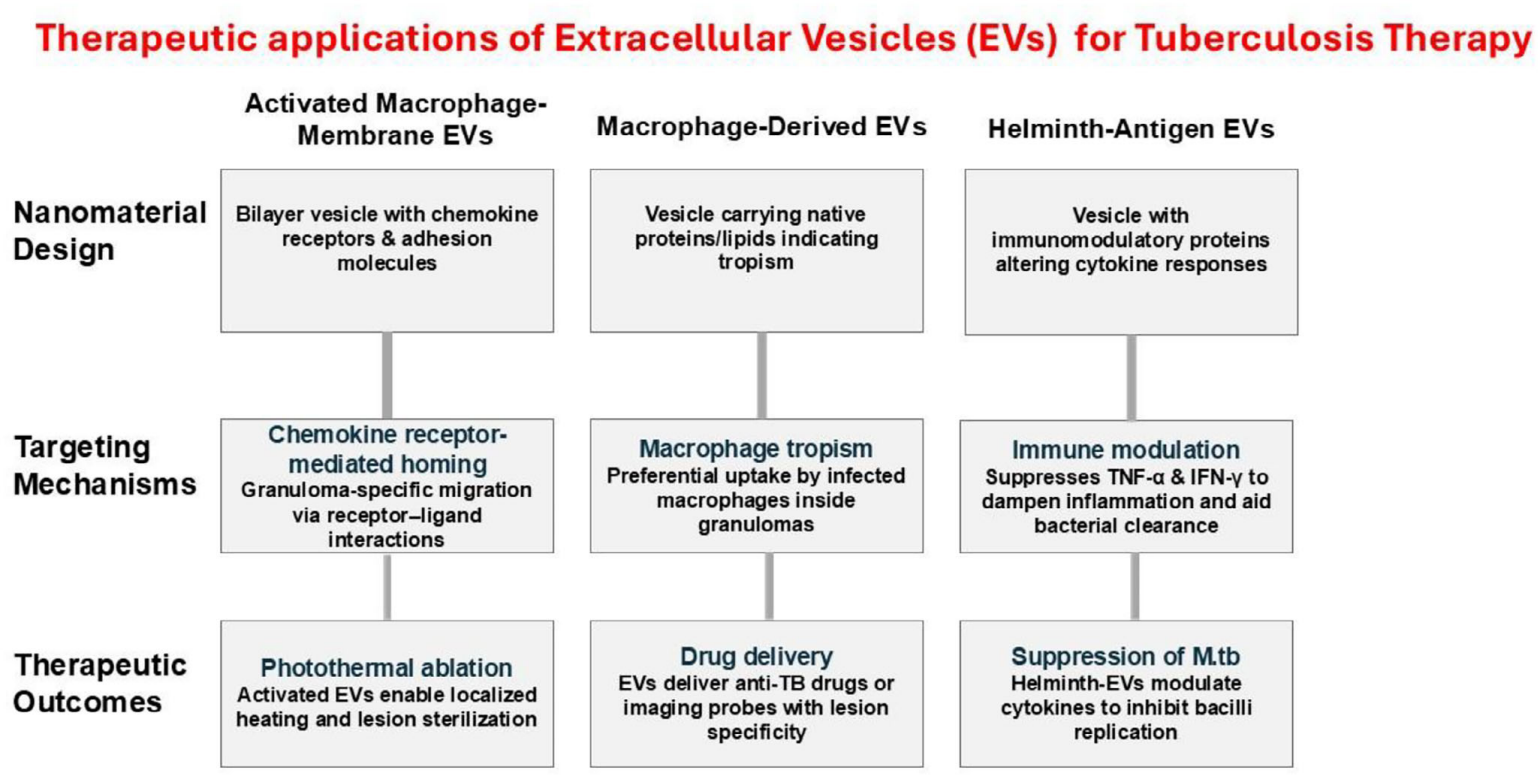
Therapeutic applications of extracellular vesicles (EVs) for Tuberculosis therapy.

Collectively, these findings indicate that biomimetic and cell‐membrane‐derived vesicles can exploit both structural mimicry and functional ligand–receptor interactions to achieve high‐fidelity granuloma targeting^[^ ^112^ ^]^ (**Figure** **10**). For LTBI, where bacilli are sequestered deep within granulomas and shielded by complex immune barriers, such active targeting approaches could significantly enhance drug delivery precision, therapeutic efficacy, and the potential for lesion‐specific imaging.

## 7 Comparative Evaluation of Active and Passive Targeting Strategies for Tuberculosis Nanodelivery

### 7.1 Limitations and Trade‐Offs

Passive targeting offers advantages in formulation simplicity, manufacturing scalability, cost‐effectiveness, and reduced immunogenicity compared to ligand‐functionalized systems. However, its lack of microenvironmental specificity can limit drug bioavailability at the site of infection.^[^ ^113^ ^]^ Active targeting, while offering greater lesion specificity, introduces complexity in synthesis, potential immunogenicity of targeting ligands, stability challenges during aerosolization, and regulatory hurdles associated with complex biologic‐nanomaterial conjugates.^[^ ^114^ ^]^

### 7.2 Integrated Approach for Optimal TB Therapy

Given the architectural and physiological complexity of TB granulomas, particularly in latent TB where bacilli are often sequestered deep within fibrotic or necrotic cores, an integrated strategy is likely to yield superior results. Pulmonary administration (passive, route‐based targeting) ensures high drug deposition at the organ level, while nanosystem engineering with ligand‐mediated targeting (active, design‐based targeting) enhances microenvironment and cellular specificity.^[^ ^115^ ^]^ Such hybrid systems could maximize therapeutic index, improve granuloma penetration, enhance macrophage targeting, and minimize systemic toxicity (**Table** **3**).

**Table 3:** Comparative analysis of passive and active targeting strategies for tuberculosis nanodelivery

Targeting strategy	Mechanism	Advantages in TB context	Limitations in TB context	Representative TB example	Refs.
Passive Targeting (Pulmonary Route)	Aerodynamic deposition in the deep lung (optimal aerodynamic diameter ≈1–5 µm); surface/charge interactions enable alveolar deposition; resident alveolar macrophages phagocytose deposited particles.	High local lung exposure; reduced systemic toxicity; non‐invasive; potential for self‐administration	Non‐uniform regional deposition; mucociliary and clearance; limited penetration into necrotic/fibrotic granuloma cores; no receptor specificity	Inhaled rifampicin powders achieve higher lung exposure vs oral dosing in preclinical models	[29, 116]
Passive Targeting (Systemic)	IV dosing leverages inflammation‐associated permeability and altered vasculature/lymphatics; EPR‐like extravasation into granulomas shown in TB models	Simple formulations; avoids ligand immunogenicity; can reach inflamed lesions.	RES uptake and broad off‐target biodistribution; limited/heterogeneous lesion access; caseum barrier	IV nanoparticles showing EPR‐like extravasation into zebrafish/mouse TB granulomas; vascular normalization improves delivery	[117, 118]
Active Targeting (Ligand‐Mediated)	Surface functionalization with ligands (e.g., mannose to CD206, antibodies, peptides) drives receptor‐mediated uptake by granuloma‐resident cells.	Increased uptake by target cells; improved lesion specificity; potential for deeper penetration when combined with size tuning	Added synthetic complexity/cost; ligand stability and potential immunogenicity; variability in receptor density and endogenous ligand competition	Mannose‐decorated nanoformulations enhance alveolar macrophage uptake/efficacy vs non‐mannosylated controls.	[41, 119]
Active Targeting (Cell‐Membrane Coated)	Nanocarriers cloaked with macrophage/dendritic‐cell membranes present native chemokine receptors for homotypic, receptor‐guided homing	Immune “self” camouflage; receptor‐guided lesion tropism; potential to navigate inflamed microvasculature	Scale‐up and batch variability; long‐term stability and aerosolization/shear sensitivity	Pre‐activated macrophage‐membrane–coated nanoparticles show enhanced granuloma homing and enable photothermal therapy in TB models.	[108, 120]
Hybrid Approach (Pulmonary and Active Targeting)	Combines route‐based organ targeting (inhalation) with design‐based ligand targeting (e.g., mannose to CD206) for microenvironment/cellular specificity	Maximises local deposition and cellular uptake; can lower systemic toxicity; synergistic targeting	Requires dual optimization (aerosol physics + ligand stability)	Pulmonary delivery of mannose‐functionalised anti‐TB nanocarriers targeting CD206⁺ alveolar macrophages	[115, 119]

## 8 Selection of Biodegradable Nanosystems

Polymer‐derived nanoparticles are colloidal structures formed from either natural or synthetic polymers. They present notable benefits when compared to other nanocarriers like liposomes, micelles, and metallic nanosystems, especially in terms of scalability and adherence to Good Manufacturing Practices (GMP).^[^ ^121^ ^]^ These polymers can either envelop an active substance within or attach it to its exterior. Among these biodegradable polymers are synthetic variants such as polyesters like poly(D, L‐lactide) (PLA), or derivatives that breakdown into lactic acid and glycolic acid like co‐polymer poly(lactide‐co‐glycolide) (PLGA), or semi‐crystalline poly‐Ɛ‐caprolactone. These materials are distinguished by their safety, minimal systemic toxicity, enhanced compatibility with biological systems, and adaptable drug‐release dynamics.^[^ ^122^ ^]^ Upon degradation, they break down into oligomers and monomers, which the body subsequently processes and expels through standard mechanisms. Research has explored the interactions between rifampicin‐loaded PLGA nanoparticles and primary macrophages infected with mycobacteria, including *M.tb* and have been rendered successful as targeted delivery.^[^ ^123^ ^]^ Antibiotics, including moxifloxacin, have been successfully combined with various polymers, such as polybutylcyanoacrylate (PBCA), for nanoparticle‐based therapy targeting *M.tb* in macrophages.^[^ ^124^ ^]^ Hence this can conclude that polymeric nanoparticle delivery is beneficial toward targeted granuloma treatment.

In addition to synthetic options, natural biodegradable polymers like chitosan, alginate, gelatin, zein, and albumin are also employed in crafting these nanoparticles.^[^ ^125^ ^]^ In 2016, Garg et al. published a study titled “Inhalable chitosan nanoparticles (CS‐NP) as antitubercular drug carriers for effective treatment of tuberculosis,” which highlighted a potential advancement in TB therapy.^[^ ^126^ ^]^ They demonstrated that drug‐loaded CS‐NPs serve as efficient carriers for targeting alveolar macrophages. This enhanced formulation led to a significant reduction in the bacillary load in the lungs and decreased cytotoxicity compared to free drugs. Additionally, it was shown that CS‐NPs not only enhance the absorption of vaccines and drugs but also extend the duration of therapeutic effects, thereby improving the effectiveness of site specific drug delivery.^[^ ^127^ ^]^

Solid lipid nanoparticles (SLNs) are delivery systems made up of solid lipids and surfactants. Their popularity has grown recently due to several advantages: they are scalable, protect drugs from degradation, improve the drug's pharmacological properties, offer consistent stability, and allow for controlled or altered drug release.^[^ ^128^ ^]^ Efforts have been made to optimize and popularize these formulations. Specifically, Costa and the team developed a standard SLN formulation of INH, and in another iteration, they enhanced it with mannose for comparative analysis.^[^ ^129^ ^]^ Interestingly, the mannose functionalized SLN demonstrated superior uptake in macrophage cells, thus showing potential in granuloma targeting.^[^ ^130^ ^]^

Dendrimers are gaining significant attention as drug delivery carriers due to their precisely controlled architecture and nanoscale size. The terminal functional groups of dendrimers exhibit greater chemical reactivity than those of other polymers, enhancing their potential for targeted drug delivery.^[^ ^131^ ^]^ Typically ranging from 1to100 nm in diameter, their intricate tree‐like designs resemble proteins, enzymes, and viruses, allowing for easy functional adjustments. Their unique copolymers possess both hydrophilic and hydrophobic units, enabling them to transport drugs with limited solubility. The complex branching of dendrimers grants them an extensive surface area relative to their volume, enhancing drug transport capabilities.^[^ ^132^ ^]^ Furthermore, characteristics like multivalence, biocompatibility, pronounced water solubility, and minimal immune system reactions highlight dendrimers as prime carriers for drug delivery, with some applications in MRI imaging.^[^ ^133^ ^]^

### 8.1 Silica Based Nanoparticles

1. Silica‐based nanomaterials constitute a versatile platform for drug delivery distinguished by their tunable morphology, surface chemistry, and porosity. They are generally classified into three main architectures:
2. Solid silica nanoparticles (SSNs) ‐ dense structures with low porosity, offering high structural stability but limited internal loading capacity.
3. Hollow silica nanoparticles (HSNs) ‐ shell‐like frameworks with large internal cavities, enabling high payload capacity, particularly for hydrophilic cargo.
4. Mesoporous silica nanoparticles (MSNs) ‐ highly ordered porous frameworks (pore diameter 2–50 nm) with exceptionally high surface areas (>800 m^2^ g^−1^) and pore volumes (>0.6 cm^3^ g^−1^), affording superior drug‐loading and controlled‐release properties.^[^ ^134^ ^]^

While all three forms have potential in biomedical applications, MSNs dominate the current literature owing to their reproducible synthesis via sol–gel or templating methods, versatile surface functionalization, and unmatched loading efficiency for small molecules, peptides, and nucleic acids.^[^ ^135^, ^136^ ^]^

#### 8.1.1 Biodegradability and Physiological Fate

Unlike polymers that undergo enzymatic or hydrolytic degradation within hours to days, amorphous silica nanoparticles, including MSNs, degrade more slowly via hydrolytic cleavage of siloxane (Si–O–Si) bonds at the particle surface.^[^ ^137^ ^]^ This process generates orthosilicic acid [Si(OH)_4_], a water‐soluble and bioavailable form of silicon naturally present in the human diet and readily eliminated through renal excretion.^[^ ^136^, ^138^ ^]^

The dissolution rate is influenced by:

1. Particle size ‐ smaller particles (<50 nm) degrade more rapidly due to higher surface‐to‐volume ratios.^[^ ^134^ ^]^
2. Porosity ‐ mesoporous frameworks dissolve faster than solid silica due to greater solvent penetration.^[^ ^139^ ^]^
3. Surface chemistry ‐ hydrophilic or organosilane‐modified surfaces can accelerate hydrolysis.^[^ ^140^ ^]^
4. pH/environment ‐ degradation is enhanced in mildly alkaline media and in acidic lysosomal compartments (pH 4.5–5.0) following macrophage uptake.

Although degradation is slower compared to organic nanocarriers, amorphous silica remains attractive for sustained‐release and chronic TB regimens due to its biocompatibility, tunable porosity, and high loading capacity.^[^ ^141^ ^]^

#### 8.1.2 Applications in Tuberculosis Nanotherapy

**Figure 11:**
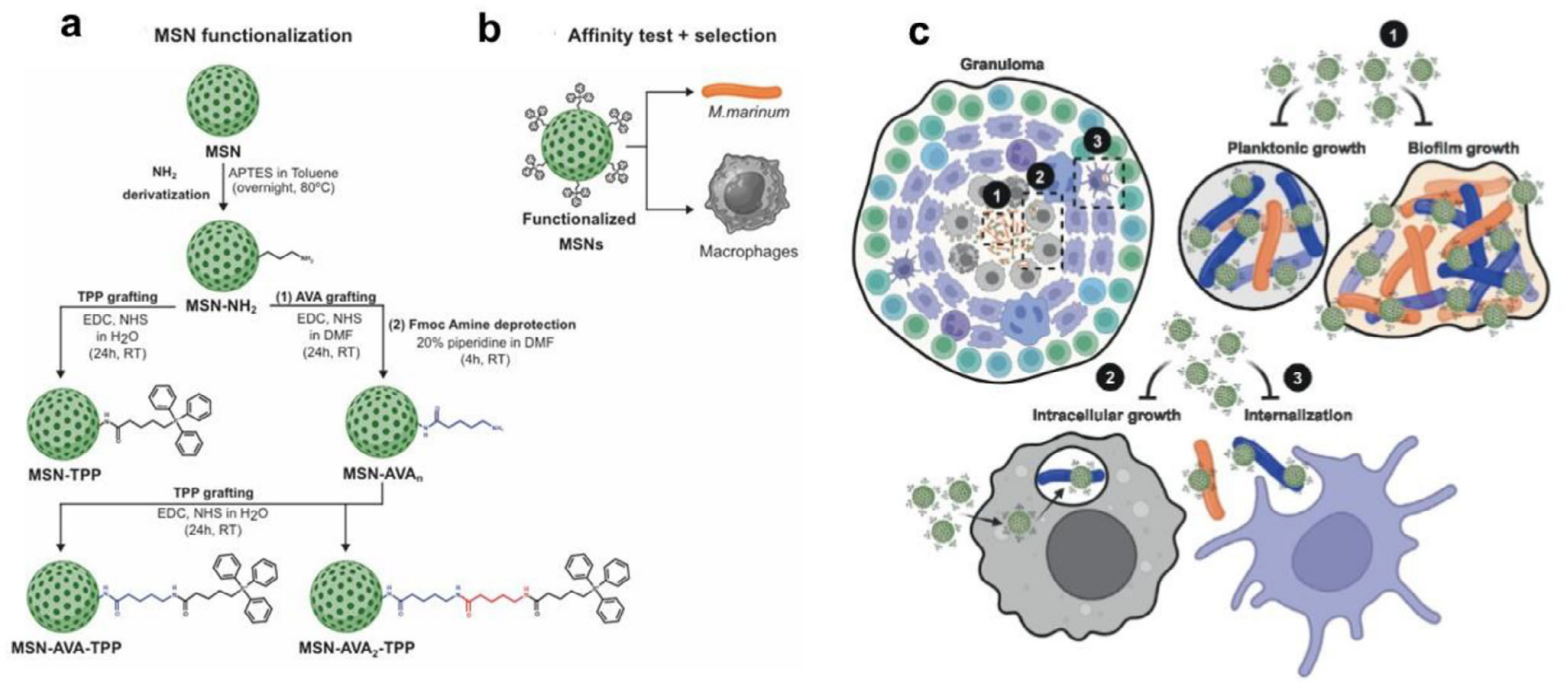
Design and intracellular efficacy of TPP‐functionalized mesoporous silica nanoparticles (MSNs) against *Mycobacterium marinum* (Mmar). a) Schematic representation of nanosystem functionalization and chemical structures of AVA–TPP ligands. b) Rationale and assay schematic of the study. c) In vitro assays demonstrate that MSN‐AVA‐TPP@DOX adheres to mycobacteria in both biofilm and planktonic states within the caseum, reducing viability of biofilm‐embedded bacilli (1). The nanosystem is also internalized into *Mmar*‐infected macrophages, reaching intracellular mycobacteria and killing them (2). Furthermore, interaction of the nanosystem with mycobacteria prevents their subsequent phagocytosis by macrophages and dendritic cells (3), thereby limiting the sporotricoid‐like lymphatic dissemination characteristic of Mmar infection.Abbreviations: TPP, triphenylphosphine; MSN, mesoporous silica nanoparticle; AVA, 5‐aminovaleric acid; DOX, doxorubicin. Images a–d reproduced under the terms of the Creative Commons Attribution (CC BY 4.0)^[^ ^145^ ^]^ license Copyright 2025, Springer Nature.

Silica‐based nanocarriers have enabled pulmonary deposition, high drug loading, and tunable release for TB therapy. For inhalation, rifampin‐loaded mesoporous silica nanoaggregates engineered by spray‐drying achieved deep‐lung aerodynamic diameters and ≈90% release within 24 h in vitro.^[^ ^142^ ^]^ Beyond simple loading, targeted/stimuli‐responsive MSNs have delivered first‐line agents: trehalose‐conjugated, photo‐functionalized MSNs released isoniazid into *M.tb* with enhanced bactericidal activity, illustrating pathogen‐binding and triggered release in infected macrophages.^[^ ^143^ ^]^ MSNs have also served as hybrid antimicrobial carriers, where AgBr‐decorated MSNs disrupted *M.tb* cell envelopes while limiting silver aggregation and toxicity.^[^ ^144^ ^]^ More recently, ligand‐engineered mesoporous silica nanoparticles functionalized with AVA–TPP ligands and loaded with doxycycline (DOX) have demonstrated potent antimycobacterial activity (**Figure** **11a**). These nanosystems not only adhered to mycobacterial surfaces and reduced bacterial burden in both planktonic and biofilm states within caseum, but also showed effective internalization into *M. marinum*‐infected macrophages, leading to the killing of intracellular bacilli (Figure 11b,c).^[^ ^145^ ^]^ Importantly, their interaction with mycobacteria further prevented subsequent phagocytosis by macrophages and dendritic cells, thereby limiting sporotricoid‐like lymphatic dissemination in zebrafish models, highlighting their translational promise for tuberculosis therapy.^[^ ^145^ ^]^

While MSNs have received the most attention, the broader family of silica‐based nanocarriers, including SSNs and HSNs, offers design flexibility for different therapeutic needs. Integrating route‐based targeting (pulmonary deposition) with design‐based targeting (ligand functionalization, stimuli‐responsive release) positions silica‐based carriers as a rational, adaptable framework for TB nanomedicine.

**Figure 12:**
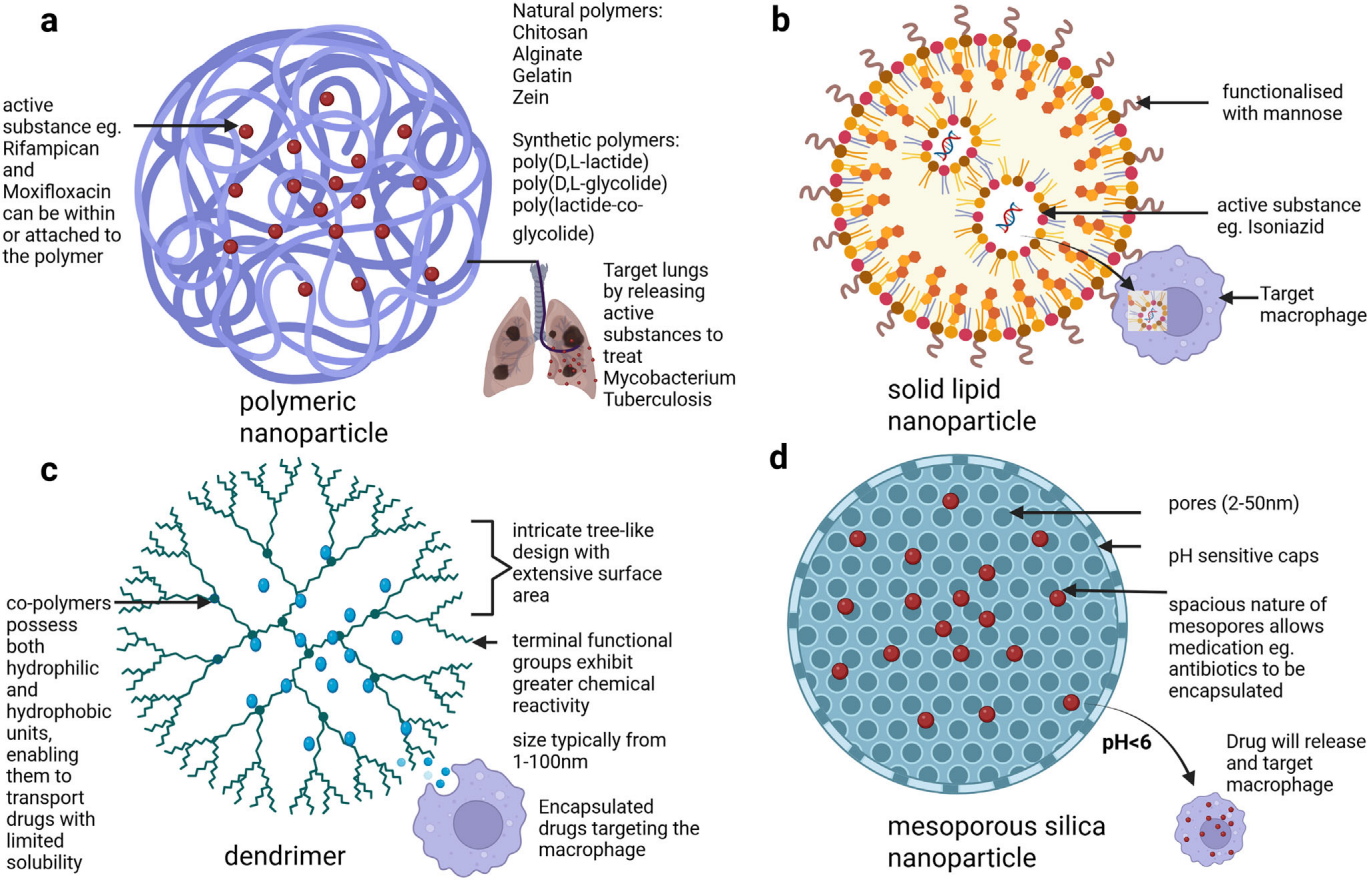
Graphical illustrations of selective nanocarriers. a) Polymeric nanoparticle, b) Solid Lipid nanoparticle, c) Dendrimer and d) Mesoporous silica nanoparticle. Image created in Biorender. Choonara, Y. (2025) https://BioRender.com/aajyns9

A summary of various nanoparticles with their respective mechanisms, advantages, and limitations is presented in (**Figure** **12**) and **Table** **4**. Each system offers unique advantages like controlled drug release, targeting, and scalability, while also presenting challenges in terms of stability, synthesis complexity, and potential toxicity.

**Table 4:** Summarizing the active targeting strategies: mechanisms, advantages, and limitations.

Nanosystem	Mechanism	Advantages	Disadvantages	Refs.
Polymeric Nanoparticles	Colloidal structures from natural or synthetic polymers that encapsulate or attach active substances.	Scalable and GMP‐compliant. Biocompatible and biodegradable, ensuring minimal systemic toxicity. Offers adaptable drug‐release dynamics for sustained delivery.	Requires optimization for desired drug release. Needs specific conditions to maintain efficacy for storage stability Can trigger immune responses or inflammation in sensitive patients.	[121, 122, 127]
Solid Lipid Nanoparticles	Composed of solid lipids and surfactants, protecting drugs from degradation and allowing controlled or altered drug release.	Scalable for large‐scale production. Protects drugs from degradation, enhancing stability. ‐ Enables sustained release, reducing dosing frequency. Enhances bioavailability.	May suffer instability without proper formulation. Some formulations may release drugs too quickly, reducing efficacy. Certain surfactants may cause cytotoxicity or irritation at high concentrations.	[130, 131]
Dendrimers	Highly branched macromolecules, 1‐100 nm in diameter. Terminal groups enable drug attachment or targeting ligands.	Dendrimers facilitate efficient drug loading. Enables controlled release and targeted delivery. ‐Multiple functional groups allow for simultaneous attachment of drugs or ligands. Biocompatible with minimal immune response.	Complex, multistep synthesis makes production expensive and time‐consuming. Mostly in preclinical or early clinical stages, limiting widespread use. Their size can limit tissue or cell penetration.	[132, 133]
Mesoporous Silica NP	Silica‐based particles with pores (2–50 nm) allowing for drug encapsulation and targeted delivery	Stimuli‐responsive drug release (e.g., pH‐sensitive). Functionalizable for targeted drug delivery. Biocompatible and stable in aqueous environments. Pores allow for the incorporation of multiple therapeutic molecules.	Requires careful optimization for preparation and functionalization. High‐dose accumulation may pose toxicity risks.	[139, 144]

## 9 Comparative Evaluation of Nanosystems for Tuberculosis Therapy

A comprehensive assessment of nanomaterial platforms is essential to inform rational design strategies for TB therapy, particularly for achieving effective granuloma targeting. The optimal carrier must balance drug‐loading capacity, release kinetics, biodegradability, targeting specificity, and safety, while remaining amenable to scalable synthesis. **Table** **5** provides a critical comparison of key nanocarrier classes discussed in this review, encompassing polymeric nanoparticles, nanostructured lipid carriers (NLCs), liposomes, dendrimers, and metallic nanoparticles, with a focus on superparamagnetic iron oxide nanoparticles (SPIONs) and mesoporous silica nanoparticles (MSNs). Where available, TB‐specific pharmacokinetic and pharmacodynamic parameters are included.

**Table 5:** Comparative evaluation of major nanocarrier platforms for tuberculosis therapy.

Nanomaterial type	Key chemical/physical properties	Common synthesis	Biodegradation pathway	Advantages (TB context)	Limitations (TB context)	Representative TB PK/PD/efficacy data	Refs.
Polymeric NPs (PLGA, Chitosan)	Biodegradable polymers; typically, 50–300 nm; surface easily modifiable (ligands/PEG/mannose)	PLGA: emulsion–solvent evaporation/spray‐drying (nano‐in‐microparticles); Chitosan: ionic gelation	PLGA: hydrolysis‐ lactic and glycolic acid; Chitosan: enzymatic degradation	Biocompatible; controlled release; chitosan is mucoadhesive; tunable degradation and surface	Lower loading for very hydrophilic drugs; risk of burst release; scale‐up and aerosolization stability to validate	Inhaled rifampicin nano‐in‐microparticles (porous aggregates) in guinea pigs achieved systemic exposure comparable to oral at ≈½ the dose; porous aggregates maintained elevated lung levels up to ≈8 h in animals.	[115]
NLCs	Solid–liquid lipid blends; ≈100–500 nm; high payload for lipophilic drugs	High shear/ultrasonication; optional ligand conjugation	Enzymatic lipolysis of lipids	High drug loading; physical stability	Release‐rate control can be formulation‐dependent; lipid oxidation; need to test aerosol/nebulization robustness.	Mannosylated rifabutin‐NLCs showed enhanced macrophage uptake and intracellular delivery with pH‐responsive release in vitro	[146]
Liposomes	Phospholipid bilayer vesicles; 50–500 nm; load hydrophilic and hydrophobic drugs	Thin‐film hydration and sonication	Phospholipid hydrolysis: RES clearance unless PEGylated	Clinically familiar platform; passive macrophage tropism after IV or inhalation; suitable for combinations	Stability and cost; rapid clearance without PEGylation; nebulization shear can destabilize	Tuftsin‐bearing rifampicin liposomes in mice achieved orders‐of‐magnitude CFU reductions vs free drug with intermittent dosing; multiple studies show improved organ CFU and survival vs free drug	[147]
Dendrimers	Highly branched, monodisperse macromolecules; precise size and high surface functionality	Divergent or convergent synthesis; surface ligand/charge tuning	Chemistry‐dependent (e.g., hydrolysis/enzymatic for biodegradable scaffolds)	Multivalent surface for targeting/antibiotic conjugation; can boost intracellular delivery	Cationic cytotoxicity risk; complex, costly synthesis; limited pulmonary data vs lipids/polymers	Report on dendrimer–antibiotic systems enhancing antimycobacterial activity and enabling targeted delivery concepts	[148]
SPIONs	Fe_2_O_3_ cores; MRI‐visible; magnetically guidable; typically, 10‐100 nm cores (often embedded in polymer)	Co‐precipitation or thermal decomposition; often co‐loaded in PLGA matrices.	Dissolution‐ Fe^3^⁺ handled by iron pathways	Theranostics (imaging and delivery); potential magnetic targeting in lung; co‐loading with TB drugs feasible	Aggregation if not stabilized; long‐term fate/safety must be evaluated; external field needed for guidance	PLGA aggregates co‐encapsulating SPIOs and bedaquiline and Q203 showed magnetic responsiveness and bactericidal activity in vitro; modeling and benchtop experiments supported magnet‐aided deep‐lung deposition	[61]
MSNs	Very high surface area; tunable 2–50 nm pores; easy surface functionalization	Sol‐gel/templating with gatekeeper modification	Hydrolysis‐ orthosilicic acid	Exceptional drug loading; engineered endosomal/acid‐triggered release; strong macrophage uptake	Slower biodegradation; pore/size uniformity critical; silica dose limits	Functionalized MSNs delivered RIF or INH to *M.tb*‐infected macrophages with superior intracellular killing vs equivalent free drug; pH‐triggered endosomal release demonstrated	[149]

Abbreviations: RIF‐rifampicin; INH‐isoniazid; RES‐reticuloendothelial system; CFU‐colony‐forming units; SPION‐superparamagnetic iron‐oxide nanoparticle.

**Figure 13:**
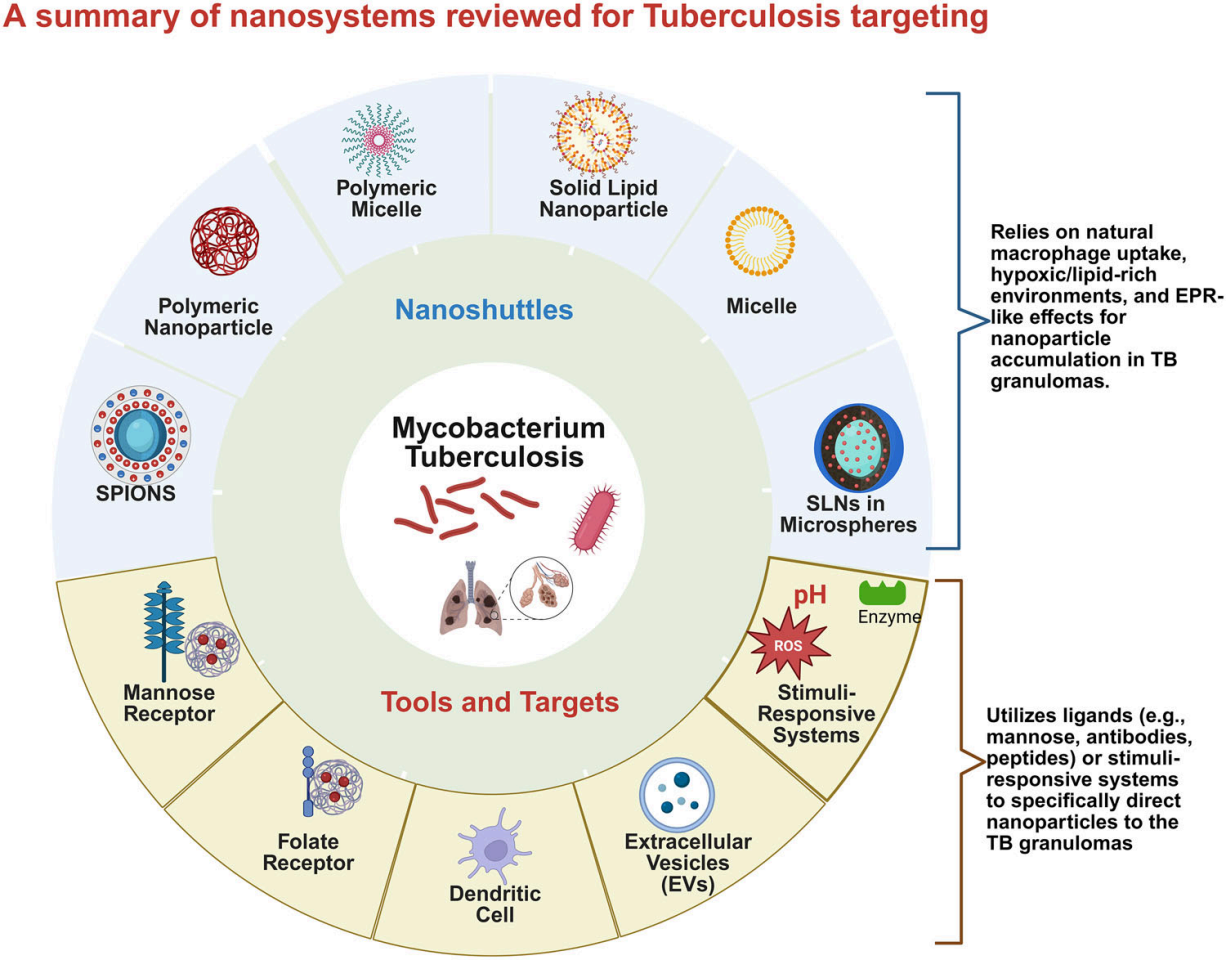
Summary of nanosystems and targeting strategies for Tuberculosis therapy. Created in BioRender. Choonara, Y. (2025) https://BioRender.com/g0a2ttz

The data in Table 5 illustrate that no single nanocarrier platform is universally optimal for tuberculosis granuloma targeting. Polymeric nanoparticles, such as PLGA and chitosan, remain versatile and clinically advanced, offering controlled release and mucoadhesion, yet their use is limited by hydrophilic drug loading challenges and risks of burst release. Lipid‐based carriers like NLCs and liposomes provide high biocompatibility and efficient pulmonary compatibility, with demonstrated efficacy in enhancing macrophage uptake and intracellular killing, though their stability under nebulization and risk of rapid clearance remain hurdles. Dendrimers excel in multivalent functionalization and precision drug delivery but face toxicity and scalability concerns. SPIONs uniquely integrate theranostic potential by combining imaging and delivery, although long‐term safety and external guidance requirements must be addressed. MSNs deliver unmatched payload and tunable release, with proven intracellular antimycobacterial efficacy, yet their slower biodegradation raises caution for chronic administration. Overall, the most promising strategy for latent TB and granuloma‐specific delivery may lie in hybrid systems that balance high loading, pulmonary stability, controlled biodegradation, and lesion‐specific functionalisation, thereby maximising therapeutic efficacy while reducing systemic toxicity. Integrating route‐based targeting with design‐based nanosystem engineering could optimise therapeutic efficacy while minimising off‐target effects (**Figure** **13**).

## 10 Challenges in Targeted Drug Delivery to TB Granulomas

Advancements in drug delivery systems for granuloma treatment have highlighted the importance of understanding the physical and chemical properties of drug carriers. These properties, such as size, shape, and surface charge, significantly influence the distribution and efficacy of drugs, especially in the complex environment of granulomas. However, there are ongoing concerns about the potential toxicity of the carrier systems, particularly in sensitive tissues such as the lungs. The surface charge, chemical makeup, and excipients used in these systems can trigger adverse effects, ranging from inflammation to more serious conditions like lung cancer.^[^ ^150^ ^]^ While biodegradable materials have helped reduce toxicity in nanosystems^[^ ^151^ ^]^ some materials still present risks, including effects on blood coagulation.^[^ ^152^ ^]^

One of the major challenges in nanoparticle‐based drug delivery for TB granulomas are premature drug release, often due to improper drug entrapment or weak adsorption within the carrier system. This premature release can result in a significant reduction in therapeutic efficacy and an increase in side effects, as the drug may be released before reaching the granuloma.^[^ ^153^ ^]^ Burst release typically occurs when a portion of the drug is weakly bound to the surface or loosely encapsulated within the delivery system, leading to rapid diffusion upon exposure to a dissolution medium.^[^ ^154^ ^]^ Factors such as poor drug entrapment efficiency, diffusion‐driven mechanisms, and the inherent properties of the drug carrier‐such as its porosity and hydrophilicity‐can further exacerbate burst release.

To address burst release in the context of granulomas, researchers have employed strategies such as modifying the carrier matrix to enhance drug encapsulation or incorporating controlled‐release coatings. Using materials like hydrophobic polymers or multi‐layer encapsulation can slow down the diffusion of the drug. For instance, polyethylene glycol (PEG)‐based coatings have been effective in providing more consistent drug release profiles and overcoming challenges like protein corona formation, which can impact the distribution of nanosystems in biological mediums, including the granuloma environment.^[^ ^155^ ^]^ Despite these advancements, the degradation of biodegradable materials and managing release kinetics remain key concerns, as the carriers eventually break down within granulomas, which can influence both efficacy and safety

Modifications to the surfaces of nanosystems, which are often necessary to improve stability and biocompatibility, also add another layer of complexity to the drug delivery process. Alterations such as attaching specific ligands or modifying the nanoparticle surfaces can dramatically affect the system's behavior in granulomas.^[^ ^156^ ^]^ Ensuring that these targeting ligands retain their binding capacity is critical for the therapeutic effectiveness of the system. Furthermore, challenges arise in maintaining the purity of nanoparticles, with residual chemicals sometimes remaining after synthesis which may affect the safety and performance of drug delivery within granulomas.^[^ ^157^ ^]^

Another promising strategy involves using triggered release systems that rely on environmental stimuli like pH, temperature, or enzyme activity within the granulomas to control drug release. This approach allows for drug release to occur primarily at the granuloma site, minimizing premature release in the bloodstream.^[^ ^57^ ^]^ This can be particularly beneficial in the treatment of tuberculosis, where localized delivery can improve therapeutic efficacy and reduce systemic toxicity. However, compatibility issues between nanocarriers and drugs persist, as many nanocarriers have specific preferences regarding the charges and hydrophobicity of the drugs they can incorporate. This often results in a low drug loading capacity, which necessitates using a larger quantity of nano‐molecules to achieve the desired therapeutic effect, ultimately introducing significant amounts of excipients into the body.^[^ ^158^ ^]^

A property‐driven design approach is essential for translating TB nanomedicine from preclinical models to the clinic. Studies indicate that precise surface charge modulation can selectively enhance accumulation in granulomatous lesions, enabling dual therapeutic and diagnostic (theranostic) applications.^[^ ^159^ ^]^ Combining this with targeting ligands and imaging functionalities allows for real‐time monitoring of drug delivery and efficacy, particularly relevant in LTBI, where bacilli are sequestered within hypoxic lesion cores.

To this end, a balanced understanding of advantages and limitations is key for platform selection, for example:

1. Polymeric and lipid‐based nanocarriers excel in biocompatibility and controlled release but may face stability issues in harsh granuloma microenvironments.
2. Inorganic systems such as mesoporous silica offer high drug‐loading capacity and tunable release but require careful biodegradation control.
3. Hybrid nanosystems can combine complementary features, such as polymer coatings on inorganic cores for responsive release and improved clearance.

By integrating physicochemical optimization with biological targeting strategies, future TB nanocarriers can be rationally engineered to overcome granuloma‐specific barriers, maximise therapeutic index, and minimise systemic toxicity‐aligning with the translational goals highlighted by the reviewer.

Translating laboratory‐designed nanosystems into clinical use for granulomas introduces several practical challenges. Issues such as high production costs, ensuring uniformity in particle size and shape, and maintaining sterility during production are ongoing obstacles.^[^ ^160^ ^]^ Additionally, the long‐term safety and effectiveness of biodegradable nanosystems have not been fully established, requiring further research and long‐term clinical studies to assess their safety profile.^[^ ^160^ ^]^ Reproducibility is also a significant issue, as consistent drug loading, encapsulation, and maintaining physicochemical properties are essential for achieving reliable and predictable therapeutic outcomes.^[^ ^161^ ^]^ Scaling up production while ensuring consistency, safety, and efficacy presents another major hurdle, compounded by the lack of regulatory guidelines for clinical application. For nanotechnology to successfully transition from the laboratory to clinical practice in granuloma treatment, optimizing production processes and developing clear regulatory frameworks are crucial steps.^[^ ^162^ ^]^

## 11 Future Direction

### 11.1 Molecular Imprinted Technologies for Targeted Drug Delivery

**Figure 14:**
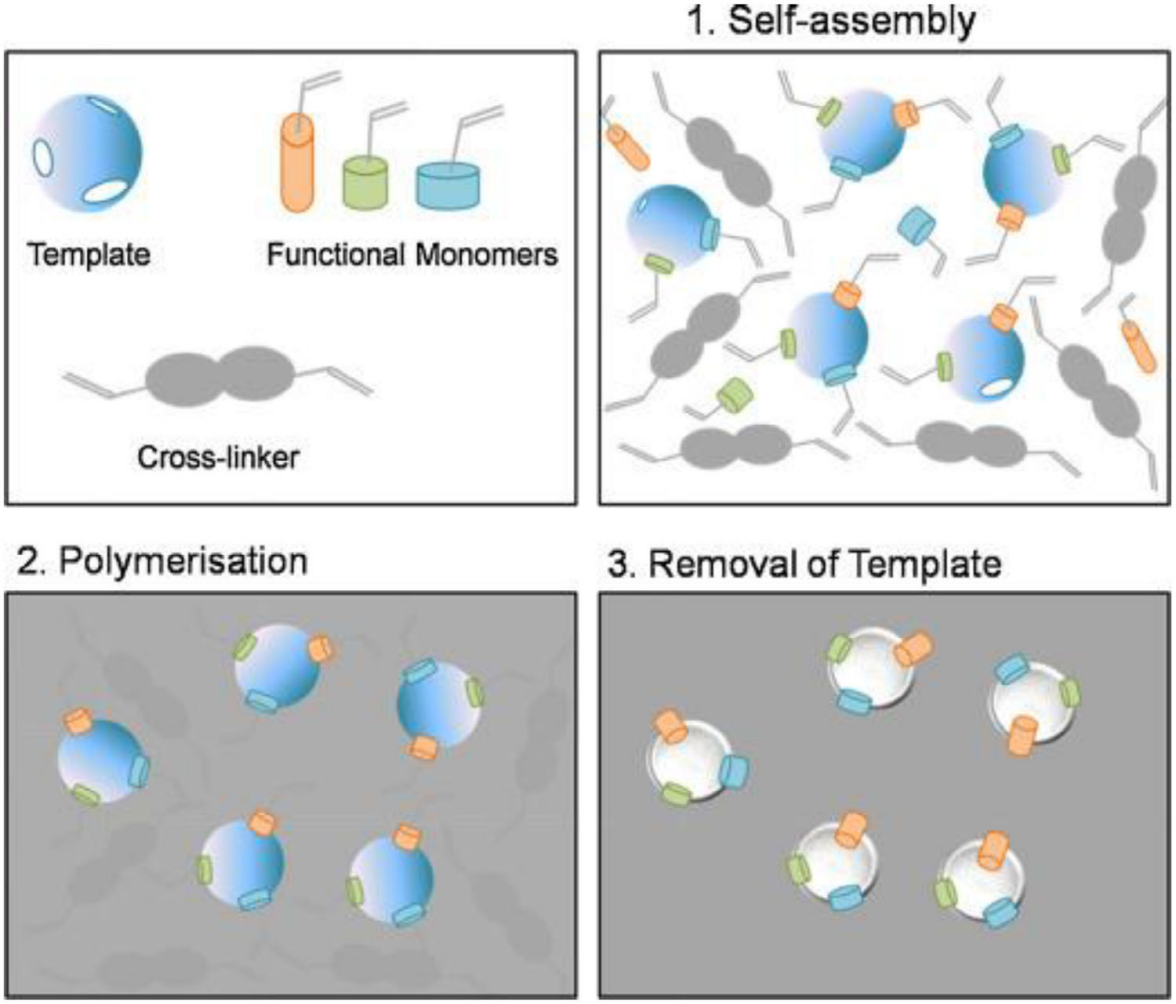
Illustrates the molecular imprinting process involving 1) The template molecule, cross‐linker, and functional monomers are dissolved in a suitable solvent, allowing for spontaneous self‐assembly, 2) Polymerization is then initiated to form a rigid polymer network, 3) The template molecule is removed, leaving behind selective binding sites that match its shape and functional groups. Reproduced with permission^[^ ^173^ ^]^ Copyright 2015, Elsevier.

Conventional drug delivery approaches often suffer from poor bioavailability and high systemic toxicity due to rapid clearance and lack of targeted distribution. These limitations reduce therapeutic effectiveness and increase the risk of adverse effects. Challenges related to systemic toxicity and reduced bioavailability are most likely due to indiscriminate delivery and swift elimination. To overcome these challenges, recent explorations have assessed various Molecular Imprinting Techniques (MIT) as innovative drug delivery systems. MIT offers a modern synthetic strategy to craft durable molecular recognition materials, replicating the specificity found in natural entities like antibodies and biological receptors.^[^ ^163^ ^]^ Molecularly Imprinted Polymers (MIP) rely on the principle of creating a complex between a specific analyte, known as the template, and a functional monomer. In the presence of excess cross‐linking agent, a 3D polymeric structure is established.^[^ ^164^ ^]^ After polymerization is complete, the template molecule is extracted, resulting in the formation of highly specific cavities that match the original molecule in shape, size, and chemical properties (**Figure** **14**). A key characteristic of molecularly imprinted polymers (MIPs) is their remarkable ability to selectively recognize and strongly bind to the target molecule used during the imprinting process. When juxtaposed with biological entities, like proteins and nucleic acids, imprinted polymers exhibit superior durability, resilience against elevated temperatures and pressures, and indifference to acidic or basic conditions, metal ions, and organic solvents.^[^ ^165^ ^]^ Moreover, they present a cost‐effective alternative with an impressive shelf life, retaining their molecular recognition abilities for extended periods even at ambient temperatures.

Until recently, numerous MIP systems have been crafted for administration via oral, intravenous, ocular, or transdermal routes. These systems have shown efficacy in treating a range of conditions, including cancer, arrhythmias, vitamin deficiencies, cardiovascular and neurological ailments, inflammatory conditions, and addiction treatments.^[^ ^166^ ^]^ Owing to their diverse administration options, significant loading potential, and specific (stereo) selectivity for intended targets, MIPs have gained traction in drug delivery. Their enhanced drug‐loading capacity, when contrasted with traditional non‐imprinted polymers, results in diminished dosages, thereby decreasing the likelihood of side effects and enhancing safety (Bodoki et al., 2021). The adaptability of MIPs positions them as valuable tools for diagnostics, imaging, drug delivery systems (DDS), or biosensors.^[^ ^167^ ^]^

Gu and colleagues developed a molecularly imprinted polymer nanoparticle (MIPNP) system for the selective delivery of a prodrug metabolite to tumors, achieving pH‐responsive release and enhanced targeting.^[^ ^168^ ^]^ Using sialic acid (SA) that is an established cancer cell marker and 5′‐Deoxy‐5‐fluorocytidine (DFCR) as co‐templates, the MIPNPs leveraged both the enhanced permeability and retention (EPR) effect and SA‐boronic acid interactions at acidic tumor sites to bind cancer cells, release the prodrug, and trigger intracellular enzymatic activation leading to cell death.

The molecularly imprinted nanocarriers demonstrated a specific ability to target the tumor site, ensuring an extended retention time and a pH‐responsive release within the tumor environment. As this MIP‐based intelligent prodrug delivery system operates independently of the liver but depends on the tumor, it offers increased specificity to cancer targets and greater versatility in choosing prodrugs. As a result, this research paves the way for innovative smart prodrug delivery systems.

In another study conducted by Parisi et al., Magnetic Molecularly Imprinted Polymers (MMIPs) were developed for controlled delivery of a 9H‐carbazole derivative in cancer therapy.^[^ ^169^ ^]^ Comprehensive in vitro studies were conducted and the crafted polymeric materials exhibited not only selective recognition and managed drug release but also a robust magnetic response. Cytotoxicity tests revealed pronounced inhibitory actions on the examined cell lines, resulting in significant growth suppression and the initiation of apoptosis when compared to controls. These findings underscore the promising role of MMIPs as magnetic‐focused drug delivery systems.^[^ ^170^ ^]^

MIPs hold potential as foundational components for advanced theranostic systems, which combine diagnostics and targeted therapy into one platform. This integration enables real‐time monitoring of treatment, guidance during surgery, and tracking of drug distribution.^[^ ^171^ ^]^ Thanks to their adaptability, MIPs can be equipped with bioimaging markers, making them useful for both therapeutic and diagnostic purposes. Specific applications include diagnosing infections like Pseudomonas aeruginosa and Helicobacter pylori by targeting their unique biomarkers using fluorescent MIPs.^[^ ^171^ ^]^ In oncology, studies have proven that MIPs can carry both anti‐cancer drugs and imaging compounds, focusing on cancer cell attributes like overexpressed glycans or surface receptors.^[^ ^171^ ^]^

The unique specificity of MIPs offers a prospective path for granuloma targeting and imaging in potential research endeavors, providing dual therapeutic and diagnostic benefits. Their precision ensures that therapeutic or imaging agents are directly channeled to granulomas, amplifying treatment outcomes and reducing unintended impacts. Moreover, their capability to stay longer at the granuloma site promotes consistent drug release and imaging.^[^ ^172^ ^]^ Given their versatility, MIPs can be customized for diverse delivery methods and granulomatous disorders. Yet, hurdles persist in fine‐tuning their specificity, ensuring biocompatibility, and circumventing undesirable biological interactions, necessitating further research and validation.

#### 11.1.1 Molecularly Imprinted Polymer Nanoparticles (MIPNPs)

MIP nanoparticles (MIPNPs) represent a notable advancement in imprinting technology. They tackle several challenges associated with MIPs crafted through bulk polymerization, such as irregular recognition site distribution, varied morphology, partial template removal, and slow mass transfers.^[^ ^174^ ^]^ Despite the complexity involved in producing MIPNPs, the advantages they bring make the detailed production process worthwhile. In essence, while traditional MIPs provided the foundational concept of molecular imprinting for selectivity and specificity, the nanoparticulate form (MIPNPs) builds on this by offering advantages related to size, delivery efficacy, and versatility, making them potentially more suitable for lung delivery applications. MIPNPs are characterized as synthetic receptors with high‐affinity demonstrating potential as imaging and therapeutic agents.^[^ ^175^ ^]^

Moczko et al., developed a study that included core‐shell MIPNPs using an innovative solid‐phase method, which utilizes immobilized templates.^[^ ^176^ ^]^ This solid phase not only assists in the creation of the nanoparticles but also protects the high‐affinity binding sites during further modifications. This technique enables the swift production, controlled separation, and purification of these specialized materials. As a proof of concept, anti‐melamine MIPNPs were coated with various features like high polarity, electro‐activity, fluorescence, and thiol groups. This versatile method offers multifunctional imprinted nanoparticles suitable for use in biosensors, diagnostics, and medical applications, presenting them as potential alternatives to natural receptors.^[^ ^176^ ^]^

Whilst MIPNPs have shown promise for targeted drug delivery, having been designed to release therapeutic agents selectively to cancer cells, enhancing efficacy while minimizing off‐target effects.^[^ ^163^ ^]^ However, comprehensive in vivo studies are essential to fully assess their safety and performance before advancing to clinical use. MIPNPs are uniquely poised for granuloma targeting due to their ability to recognize and bind to specific molecules with high selectivity and affinity. Given that granulomas form in response to persistent antigens or pathogens, MIPNPs can be custom designed to recognize and bind to specific antigens or molecules associated with granulomas. This molecular recognition capability ensures targeted delivery of therapeutic or diagnostic agents directly to the granuloma site, potentially enhancing treatment efficacy and imaging accuracy. Furthermore, MIPNPs exhibit high stability, resistance to biodegradation, and can be engineered to release their drug payload in response to specific environmental cues found within the granuloma such as pH or enzymatic activity. These characteristics could offer prolonged retention within the granuloma and sustained release, making them promising candidates for granuloma‐targeted therapies and diagnostics.

### 11.2 Immunotherapy for Targeted Drug Delivery

While the term “immunotherapy” is traditionally linked to cancer treatment, as evident from the bulk of published research, the strategy is quickly broadening its relevance to a wider array of diseases^[^ ^177^ ^]^ This expansion prominently features therapies that bolster T cell functionality and ligand‐based treatments aimed at neutralizing or eradicating afflicted cells. Emerging therapies like immune checkpoint inhibitors and antibody‐based treatments are showing potential. T cell modifications, especially with CAR T cells, have revolutionized adoptive therapies. Moreover, successes with antibody conjugates in the cancer domain are hinting at parallel strategies for infectious diseases, encompassing HIV and various microbial infections. Consequently, we can anticipate a surge of novel therapeutic agents and increased pre‐clinical investigations across different disease paradigms.^[^ ^177^ ^]^ Immunotherapies leverage or regulate the body's immune mechanisms to address various diseases. In the context of conditions with granulomas, the therapeutic strategy revolves around modulating the immune system to either inhibit the development of granulomas or aid in resolving the existing ones. The use of checkpoint inhibitors has emerged, these medications activate the immune response to combat specific ailments. In conditions such as sarcoidosis, checkpoint inhibitors, including PD‐1 or CTLA‐4, which are under investigation for cancer therapies‐might control hyperactive immune reactions and deter the development of granulomas.

These therapeutic agents are designed to enhance the immune system's ability to combat particular diseases. In cancer treatment, immune checkpoint inhibitors, especially those targeting PD‐1 (Programmed Death‐1) and CTLA‐4 (Cytotoxic T‐Lymphocyte‐Associated Protein 4), have emerged as promising strategies for improving outcomes across a range of malignancies.^[^ ^178^ ^]^ Their mechanism of action involves unlocking the “brakes” on the immune system, allowing for a more robust immune response against tumors.^[^ ^162^ ^]^ Given this capability, there's a growing interest in understanding whether these inhibitors could be repurposed for diseases like sarcoidosis. By modulating an overzealous immune system, they might prevent the unchecked immune responses that lead to granuloma formation. It's worth noting, however, that while the promise of checkpoint inhibitors is considerable, their application outside of oncology remains a burgeoning area of research, with safety and efficacy yet to be fully established.^[^ ^179^ ^]^

Gene therapy represents an innovative frontier in medicine, offering the potential to target and alter specific genetic sequences to treat various conditions.^[^ ^180^ ^]^ In the context of granulomas, this approach could be harnessed to both modulate granuloma formation and promote the resolution of existing granulomas. Granulomas often form as a result of chronic inflammation; therefore, introducing or silencing genes associated with inflammatory cytokines or other inflammatory markers could potentially modulate the body's inflammatory response. For example, the introduction of genes that promote the production of anti‐inflammatory cytokines, such as IL‐10, could help prevent or reduce granuloma formation.^[^ ^181^ ^]^ A study by Wong et al. (2020) highlighted the critical role of IL‐10 in preserving the immune balance required for granulomas to manage bacterial load and disease progression in *M.tb* infection.^[^ ^182^ ^]^ Further studies have shown that IL‐10 significantly impacts the host's ability to control bacterial load, particularly after 3 months of infection.^[^ ^183^ ^]^ Additionally, gene therapy could alter the activation of molecular pathways such as NF‐κB or MAPK signaling, which regulate immune cell function in granulomas. By manipulating these pathways, gene therapy could reduce the recruitment of inflammatory immune cells, promote macrophage polarization toward a pro‐resolving M2 phenotype, and enhance the differentiation of regulatory T‐cells (Tregs) that help dampen chronic inflammation.^[^ ^184^ ^]^ These targeted interventions could not only improve granuloma resolution but also reduce tissue damage and fibrosis, ultimately leading to better disease outcomes.

However, several challenges must be addressed for successful gene therapy in granulomas. Targeting specific tissues, especially in chronic conditions like tuberculosis, remains a significant hurdle. The granulomatous tissue often exhibits irregular vasculature and immune cell infiltration, which complicates efficient gene delivery and targeting. Furthermore, overcoming immune system barriers, such as the activation of the body's immune response to foreign vectors, remains a major challenge.^[^ ^185^ ^]^ Safety concerns also include the risk of off‐target effects, where gene editing may inadvertently affect non‐target genes, and the potential for long‐term impacts, such as immune system dysregulation or tumorigenesis due to the introduction of foreign genetic material. These factors must be carefully evaluated and mitigated to ensure that gene therapy is both effective and safe for patients with granulomatous diseases.

The Clustered Regularly Interspaced Short Palindromic Repeats (CRISPR) system, along with its associated proteins (most notably Cas9), represents one of the most exciting breakthroughs in modern biology. CRISPR‐Cas9 allows for precise genome editing, which has wide‐ranging implications in both research and therapeutic contexts. The CRISPR‐Cas9 system provides a forefront solution to potentially address ailments marked by granulomas. In afflictions such *M.tb*, this genetic tool might be designed to directly counteract the causative agent by impeding its essential genetic components, potentially reducing the onset of granulomas.^[^ ^186^ ^]^ Additionally, the body's immune reactions that culminate in granuloma development could be fine‐tuned with CRISPR by modifying genes linked to macrophage response and T‐cell activities.^[^ ^187^ ^]^ The tool also offers promise in recalibrating the inflammation balance by influencing genes in both pro‐inflammatory and anti‐inflammatory circuits.^[^ ^188^ ^]^

Delivery systems are crucial for the success of CRISPR‐based gene therapies, especially in targeting granulomas. Advanced nanoparticles and viral vectors have emerged as the most promising methods for efficient delivery to granulomatous regions. Nanoparticles can be engineered for size and surface modifications to enhance uptake by immune cells in granulomas, while viral vectors such as adeno‐associated viruses (AAVs) offer high specificity for targeted delivery.^[^ ^189^ ^]^ When comparing these methods, nanoparticles offer potential for sustained release and lower immunogenicity compared to viral vectors, which may elicit immune responses or cause unintended integration into the host genome. However, both systems face challenges in ensuring precise delivery to granulomatous tissue and avoiding off‐target effects.^[^ ^190^ ^]^

While CRISPR‐based therapies hold promise, more clinical evidence is required to establish their effectiveness for granulomatous diseases. There are a few ongoing clinical trials evaluating gene therapies for inflammatory conditions like Crohn's disease and rheumatoid arthritis, which share common inflammatory pathways with granulomatous diseases.^[^ ^191^ ^]^ These studies show early promise in manipulating immune responses, but their direct application to granulomatous inflammation remains in its infancy. Furthermore, ethical and practical considerations, including the potential for germline editing and long‐term safety, must be addressed. The implications of genetically modifying immune cells or other tissues raise concerns about unintended effects, such as immune dysregulation or tumorigenesis, and regulatory approval for clinical applications of gene therapy is still a challenge.^[^ ^192^ ^]^ Thus, while CRISPR presents a revolutionary approach, its clinical translation must carefully navigate these ethical, safety and regulatory hurdles.

### 11.3 Antimicrobial Peptides

Among the emerging therapeutic strategies, antimicrobial peptides (AMPs) have gained considerable attention due to their dual function as both antimicrobial agents and immunomodulators. These naturally occurring peptides can selectively target infected macrophages and inflamed tissues, allowing for enhanced drug accumulation within granulomatous lesions. Furthermore, their ability to disrupt bacterial membranes and penetrate dense tissue structures makes them suitable candidates for integration into nanoparticle‐based delivery systems. Lassomycin, a cyclic AMP has shown potent bactericidal activity against Mycobacterium tuberculosis, including multidrug‐resistant strains. Its mechanism‐targeting the ClpC1 protease complex‐renders it uniquely effective in disrupting bacterial survival pathways. When delivered via AMP‐functionalized nanocarriers, Lassomycin's therapeutic potential can be significantly amplified, as such systems facilitate deeper granuloma penetration and sustained drug release.^[^ ^193^ ^]^ This integrated approach offers a promising avenue for enhancing treatment efficacy while minimizing off‐target effects, especially in persistent TB infections where conventional therapies often fail to reach the bacterial niche effectively.

## 12 Conclusion

Biodegradable nanosystems designed for specific drug delivery to treat granulomas have demonstrated considerable potential. Granulomas, which are the body's organized response to sustained inflammation, require precise treatment delivery to ensure therapeutic efficacy. Leveraging drug targeting strategies can reduce the drug dosage required to achieve therapeutic effects, streamline the administration process, lower treatment costs, and minimize side effects by reducing non‐specific drug accumulation. With their reduced toxicity and improved adaptability, biodegradable nanosystems offer an effective approach. However, significant challenges remain, including achieving thorough penetration into granulomatous tissue, controlling drug release, and navigating the complex granuloma environment.

Specific research gaps include exploring advanced targeting ligands or homing peptides that could increase the specificity of nanosystems for different types of granulomatous tissues, such as those seen in tuberculosis vs sarcoidosis. Additionally, future studies should focus on developing imaging and monitoring techniques to track nanosystem penetration and distribution in real‐time, providing deeper insights into the retention and behavior of these systems within dense granulomatous structures. The need for innovation in materials science is critical. Stimuli‐responsive polymers or biodegradable hybrid materials could create nanosystems that react to the specific pH or enzyme profiles of granulomas, ensuring more controlled and sustained drug release. Research into multi‐functional “theranostic” nanosystems that combine therapeutic and diagnostic functions might further optimize treatment efficacy by simultaneously treating and monitoring disease progression.

To facilitate clinical translation, addressing regulatory requirements is essential, especially concerning consistent production quality and the stability of these nanosystems. Scalable microfluidic synthesis techniques for nanoparticle production could help standardize the manufacturing process, enabling better consistency in batch production and aiding regulatory approval. Long‐term safety and ethical considerations are paramount, with further research needed into the biocompatibility of biodegradable nanosystems and the potential for immune responses triggered by residual nanoparticles or their breakdown products over time. Ethical concerns, such as informed patient consent, should also be carefully considered, particularly for systems that may have prolonged retention in the body.

The broader implications of these advancements are significant, particularly for populations in low‐resource settings that bear a high burden of granulomatous diseases. Nanosystems could reduce systemic toxicity and treatment costs, making therapies more accessible and sustainable, especially in regions with limited healthcare infrastructure. These advancements have the potential to revolutionize treatment approaches by minimizing dosage requirements and side effects, offering better health outcomes in underserved populations. Sustained collaborative research efforts, combined with clinical evaluations, are essential for unlocking the full potential of these innovative treatments for granuloma‐related diseases.

## Conflict of Interest

The authors declare no conflict of interest.

## Author Contributions

S.R.S. contributed to the study through conceptualization (equal), data curation (equal), formal analysis (equal), investigation (lead), methodology (lead), software (lead), writing – original draft (lead), and writing – review and editing (equal). M.M.M. was involved in conceptualization (equal), formal analysis (equal), investigation (equal), supervision (equal), validation (equal), and writing – review and editing (equal). T.M. contributed to conceptualization (lead), data curation (equal), formal analysis (lead), funding acquisition (lead), investigation (equal), project administration (lead), supervision (lead), validation (lead), visualization (supporting), and writing – review and editing (equal). Y.E.C. participated in conceptualization (equal), data curation (equal), formal analysis (equal), funding acquisition (lead), investigation (equal), project administration (lead), supervision (lead), validation (lead), visualization (supporting), and writing – review and editing (equal).
